# Expert Review on the Use of Methylphenidate in Children and Adult With Epilepsy and Attention Deficit Hyperactivity Disorder

**DOI:** 10.31083/RN52291

**Published:** 2026-10-29

**Authors:** Víctor Soto Insuga, María Eugenia Russi, Elena González Alguacil, Javier López Pisón, Sergio Aguilera Albesa, Ignacio Málaga Dieguez, Carmen Fons

**Affiliations:** ^1^Unidad de Neurología, Hospital Niño Jesús, 28009 Madrid, Spain; ^2^Área de Neurología Pediátrica y Neurofisiología. Hospital Sant Joan de Déu. Institut de Recerca Sant Joan de Déu. EpiCARE Coordinator Center, 08950 Barcelona, Spain; ^3^Servicio de Pediatría, Hospital Miguel Servet, 50009 Zaragoza, Spain; ^4^Unidad de Neuropediatría, Servicio de Pediatría, Hospital Universitario de Navarra, 31008 Pamplona, Spain; ^5^Unidad de Neurología Pediátrica, AGC de Pediatría, Hospital Universitario Central de Asturias, 33011 Oviedo, Spain

**Keywords:** epilepsy, methylphenidate, neurology, paediatrics, attention deficit hyperactivity disorder (ADHD)

## Abstract

Attention deficit hyperactivity disorder (ADHD) is a comorbidity affecting 12%–29% of individuals with epilepsy, significantly impacting learning, psychosocial development, behaviour, and quality of life. Moreover, ADHD and epilepsy share a bidirectional relationship. Although methylphenidate has been proven effective in alleviating ADHD symptoms, its use remains limited in individuals with epilepsy due to outdated concerns—dating back over 20 years—that psychostimulants could increase seizure frequency. This manuscript presents a narrative review of the scientific literature published between January 2000 and May 2026 regarding the use of methylphenidate in patients with epilepsy and ADHD. Based on the available evidence, a panel of paediatric neurologists from five Spanish tertiary hospitals with expertise in epilepsy and neurodevelopmental disorders reached an expert consensus and developed a series of practical recommendations for the assessment and management of these patients. This expert group issued several recommendations, including the proactive screening of ADHD symptoms in all children and adults with epilepsy from the onset of the condition. Current evidence has not consistently demonstrated an increased seizure risk associated with methylphenidate in patients with epilepsy, although available data remain limited for some epilepsy subgroups. Furthermore, undertreatment of ADHD is associated with poorer patient outcomes. Methylphenidate may be considered in children and adults with epilepsy when clinically indicated, following optimization of epilepsy management, associated comorbidities, and behavioural and psychosocial interventions. Treatment decisions should be individualized, particularly in patients with active epilepsy, drug-resistant epilepsy, recent seizure worsening, or developmental and epileptic encephalopathies.

## 1. Introduction 

Attention deficit hyperactivity disorder (ADHD) is currently regarded as one of the most common neurodevelopmental disorders, and is a common presenting complaint in paediatric neurology practice. It is a neurobiological disorder which is characterised by difficulties in regulating activity levels (hyperactivity), inhibiting thoughts and/or behaviours (impulsivity) and maintaining attention while engaging in tasks that require cognitive effort (inattention) [1,2,3,4,5]. Its prevalence worldwide is estimated at around 5–7% in children, and 1–5% in the adult population [6,7]; and at 6.8% in Spain’s paediatric population [8].

Meanwhile, epilepsy is defined as a brain disorder characterised recurring epileptic seizures, which is associated with neurobiological, cognitive-behavioural and social consequences, and is a very common presenting complaint in neuropaediatrics. Its rate of prevalence in Europe is 0.7% of the population, affecting around six million people (between 300,000 and 400,000 in Spain), with 400,000 new cases each year and an incidence worldwide of 61.44 per 100,000 people per year [9]. Children with epilepsy have a high prevalence of psychiatric and neurological comorbidities (including learning difficulties, mood disorders, anxiety, autism spectrum disorder (ASD) and cognitive impairments). Among all these conditions, ADHD is the most common comorbidity in children with epilepsy [10].

According to several recent meta-analyses, ADHD prior to the onset of epilepsy is common in children, with an estimated prevalence of 12%–39%, which is a risk three to five times higher than in the population without epilepsy [11,12,13,14,15,16,17,18]. The prevalence of ADHD is even higher in children with drug-resistant epilepsy, accounting for as many as 70% of cases [19]. However, this relationship is two-way [20], meaning that epilepsy may also be present in 3.4% of people with ADHD (2.53–4.21%) [11,21]. Estimates suggest that as many as 20–50% of children with epilepsy have subclinical symptoms of ADHD [22]. This very common association is due to the fact that both epilepsy and ADHD share common neurobiological mechanisms, including aberrant frontocerebellar and frontostriatal neural networks, alterations in ion channel function, and imbalances in neurotransmission [23] related to genetic factors and to the epileptiform activity itself, and epigenetic factors, which affect neuronal plasticity and regeneration and which may contribute to both the neuronal hyperexcitability typical of epilepsy, and to the attentional and executive function impairments observed in ADHD [11,18]. Some specific genetic neurodevelopmental disorders are associated with a high prevalence of both conditions. These include dystrophinopathies, in which higher prevalences of both epilepsy and symptoms of ADHD and other cognitive and behavioural disorders have been reported, as well as Noonan syndrome and other RASopathies, in which alterations in the signalling pathways involved in brain development may contribute to the onset of neurodevelopmental disorders and epilepsy [24].

While there does not appear to be any significant difference between the characteristics of ADHD in children with epilepsy and in the population without epilepsy [25], some authors report a higher proportion of inattentive ADHD in cases with comorbid epilepsy [26,27]. Furthermore, while ADHD predominantly affects boys rather than girls, with a ratio of between 2:4 and 4:1 (depending on whether the studies are clinical or observational), this gender difference has not been reported in children with epilepsy [28,29,30].

### Rationale for the Development of Expert Recommendations

As well as being the most frequently reported comorbidity in children with epilepsy, ADHD has a negative impact on various aspects of an individual’s life, ranging from their learning and academic performance to areas related to social cognition, executive functions, mood and anxiety. This is one of the most important factors contributing to a poorer quality of life for both children with epilepsy and their relatives [31,32]. Furthermore, people with epilepsy often experience symptoms of inattention, motor restlessness and impulsivity that persist into adulthood, with an estimated 15–18% of adults with the condition continuing to experience these symptoms, and an estimated prevalence of ADHD which is more than double that of people without epilepsy. For all these reasons, the specific treatment of comorbid ADHD based on a holistic approach to the management of patients with epilepsy is crucially important [33,34].

There has been a consensus for many years that the therapeutic approach for all patients with ADHD should be multimodal; i.e., it should include a combination of psycho-educational interventions, remedial support and educational assistance, together with cognitive-behavioural therapy, psychoeducation, psychosocial support in the child’s environment and pharmacological treatment from the age of six years old onwards [35]. As regards the type of pharmacological treatment, numerous controlled studies have demonstrated the efficacy of both psychostimulant and non-psychostimulant drugs. However, there is currently a consensus that central nervous system (CNS) stimulants, or psychostimulants, such as methylphenidate and lisdexamphetamine, are the first-line treatments for ADHD. These medicines have a larger effect size, and fewer of them are needed for treatment than non-psychostimulants; stimulants are among the most widely used drugs for the treatment of ADHD, due to their efficacy and safety. Within this pharmacological group, methylphenidate is regarded as the first-line treatment for ADHD in Spain, with an estimated efficacy of around 65%–85% [35].

Despite the importance of identifying ADHD at an early stage in patients with epilepsy, it often remains underdiagnosed, and these patients therefore do not receive appropriate pharmacological treatment [26]. This is partially due to long-standing concerns about the safety of using psychostimulants in patients with epilepsy, as it was thought that they might lower the seizure threshold and increase related morbidity. However, recent studies have shown that psychostimulants such as methylphenidate can be used safely, without increasing the frequency of seizures in patients with epilepsy. Given the lack of a specific treatment for ADHD associated with epilepsy, a review of the scientific evidence available, with recommendations based on the evidence and expert opinion are needed.

In order to address this issue, a group of paediatric neurologists at five Spanish hospitals (specializing in epilepsy and neurodevelopmental disorders, and focusing particularly on ADHD in children and adolescents), conducted a review of the evidence published between January 2000 and May 2026, which involved an analysis of more than 140 articles. They subsequently drew up a series of recommendations, which are presented in Fig. 1.

**Fig. 1.**
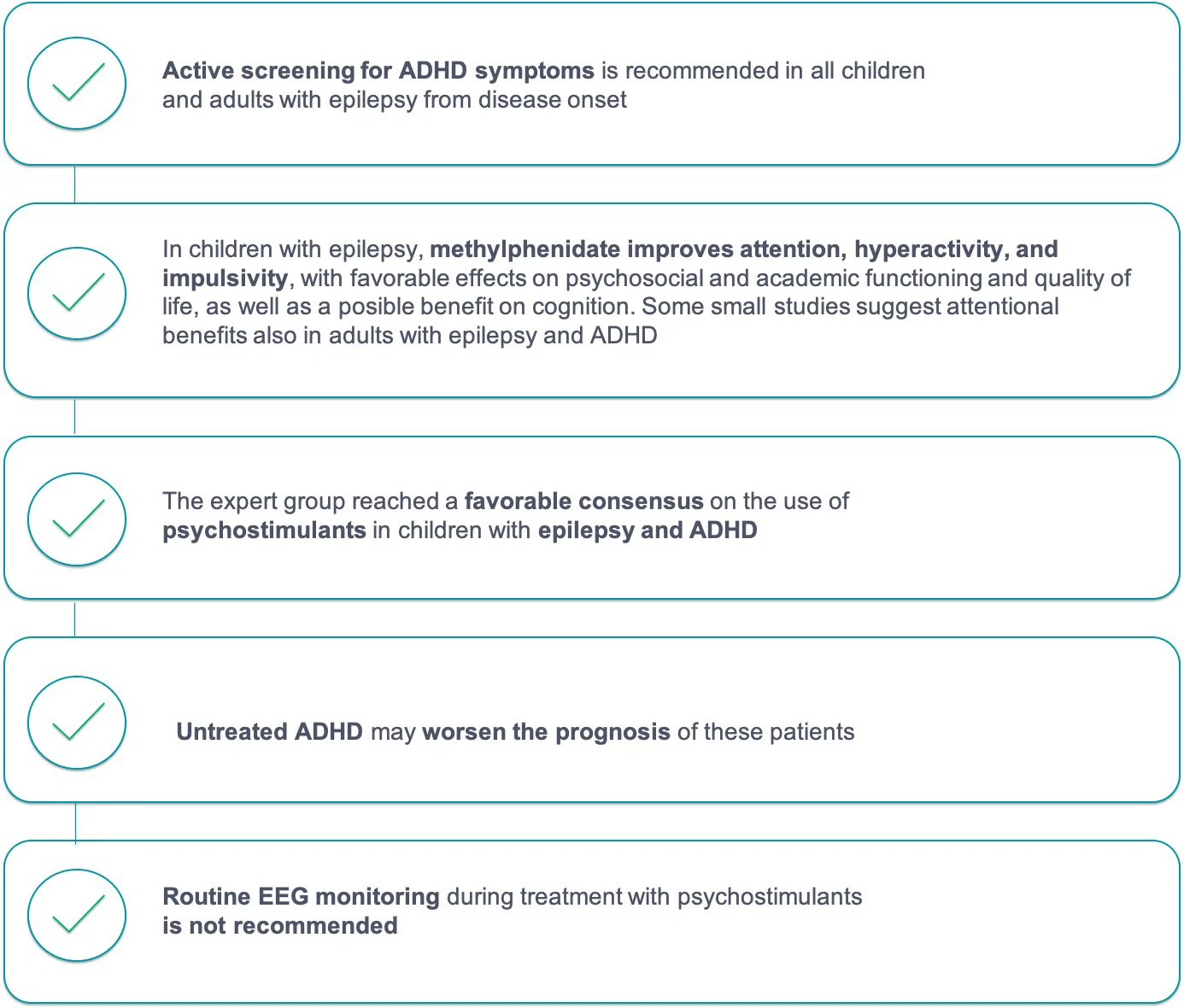
**Recommendations by the group of experts on the management of patients with ADHD and epilepsy**. ADHD, attention deficit hyperactivity disorder; EEG, electroencephalogram.

This review consists of a narrative review of the scientific literature published between January 2000 and May 2026 on the use of methylphenidate in patients with epilepsy and ADHD, supplemented by the development of practical recommendations based on the consensus of a group of experts. The literature search was carried out primarily in PubMed/MEDLINE, and was supplemented by a manual review of the references in the articles identified, and of relevant documents published by international scientific societies. Various combinations of terms relating to ‘epilepsy’, ‘attention deficit hyperactivity disorder’, ‘ADHD’, ‘methylphenidate’, ‘stimulants’, ‘psychostimulants’, ‘children’ and ‘paediatric’ were used. This search strategy identified more than 140 relevant articles, which were selected on the basis of their clinical and scientific relevance to the objectives of the document, and included observational studies, clinical trials, systematic reviews, meta-analyses, consensus documents and clinical practice guidelines. As the aim of this review was to produce a narrative review containing practical recommendations based on clinical experience, the methodology usually used for a systematic review was not applied. Consequently, no formal assessment of the risk of bias in the studies included was performed, the quality of the evidence was not graded using standardised systems (such as Grading of Recommendations Assessment, Development and Evaluation (GRADE)), and a quantitative meta-analysis was not conducted. The articles were selected based on their clinical and scientific relevance, which was supplemented by the expert judgement of the working group.

After the scientific literature identified had been reviewed, a panel of experts was formed, consisting of paediatric neurologists from five Spanish hospitals with experience in epilepsy and neurodevelopmental disorders. The consensus process involved three online meetings, during which the available evidence was reviewed and discussed. Proposals for practical recommendations were drawn up and were discussed following this review, and they were subsequently amended and refined in an iterative process until all the participants reached agreement. The final recommendations reflect the consensus of the group of experts, which was based both on the available scientific evidence and on the authors’ clinical experience, as set out in Fig. 1.

## 2. Risk Factors and Comorbidities of ADHD in People With Epilepsy

There are several risk factors with statistically significant links to the development of ADHD in patients with epilepsy and common comorbid symptoms that can exacerbate the symptoms of ADHD. It is therefore essential to be able to identify these factors. Cognitive disorders, a family history of ADHD and epilepsy, refractory seizures, epileptiform abnormalities, the type of epilepsy, anticonvulsant medications, psychiatric disorders and sleep disorders are basic factors that must be taken into account in order to ensure that these patients are managed appropriately.

### 2.1 Cognitive Disorders

The presence of an intellectual disability has been identified as an independent risk factor for ADHD in children with epilepsy [27]. Children with epilepsy and an intellectual disability are consequently at greater risk of also presenting ADHD. In a study of 85 children with epilepsy, ADHD was present in 20% of patients with normal cognitive function, compared to 59% of those with an intellectual disability [36].

### 2.2 Family History of ADHD and Epilepsy

The global heritability rate of ADHD is estimated at 76% [27]. However, there is less evidence on this issue in the population with epilepsy, although in one small study of 16 children with ADHD and epilepsy, 50% of the mothers met the criteria for ADHD [37]. According to a recent review of 46 studies, having a family member with epilepsy was associated with a 2.58-fold increased risk of developing ADHD [27].

### 2.3 Refractoriness and Type of Epilepsy

Although ADHD tends to be more prevalent in refractory epilepsies [38], it is also a highly common comorbidity in self-limited epilepsies, which do not usually require many lines of anti-seizure treatment. These include self-limited epilepsy with centro-temporal spikes (SeLECTS), in which ADHD is usually present in more than half of cases (estimated at 60%) [39], and childhood absence epilepsy, in which the prevalence of ADHD ranges from 26–57% [27,40].

Whilst ADHD may be present in epilepsies affecting different brain regions, it appears to be more common in those affecting the frontal lobe, as the prefrontal cortex and the inferior frontal gyrus are key areas for the correct development of executive functions [28].

### 2.4 Anticonvulsant Medications

Although any anticonvulsant medication with the potential to affect cognition (particularly GABAergic drugs) should be considered a factor that may exacerbate ADHD symptoms, the only anticonvulsant for which there is Grade A evidence that it may worsen inattention symptoms is valproic acid, with treatment being discontinued in up to 25% of cases [41]. Various reviews have also shown that polypharmacy is associated with a higher probability of developing ADHD [27].

Current evidence suggests that new-generation drugs such as brivaracetam, perampanel and cenobamate generally have a relatively neutral cognitive profile. Furthermore, some treatments that have recently been included in the management of some epileptic and developmental encephalopathies, such as cannabidiol and fenfluramine, have been shown to have beneficial effects other than their anticonvulsant properties, on aspects related to alertness, social interaction and behaviour. However, the evidence available is still insufficient for specific recommendations regarding its potential impact on the core symptoms of ADHD [42].

### 2.5 Psychiatric Disorders

Mood disorders, anxiety, conduct disorder and behavioural disorders (including oppositional defiant disorder) are highly prevalent both in people with epilepsy and in children with ADHD. Early identification is therefore recommended [41].

### 2.6 Sleep Disorders

It is estimated that up to 84% of children with epilepsy have some form of sleep disorder, the most common of which include: insomnia (13–31%), hypersomnia (10–47%), parasomnias (52%) and sleep apnoea syndrome (10–30%). Likewise, 25–70% of children with ADHD experience frequent sleep disorders [43].

### 2.7 Epileptiform Abnormalities

Interictal epileptiform abnormalities are common in children with epilepsy and ADHD, with estimates suggesting that up to 51% present interictal epileptiform abnormalities in a sleep electroencephalogram (EEG), compared with an incidence of 1.4%–6.5% of epileptiform abnormalities in children without epilepsy [41]. However, several studies have concluded that there is currently no evidence of a statistically significant relationship between interictal EEG changes and the severity of attention deficit [27].

### 2.8 Other Factors

There is as yet no consensus, or the studies are contradictory, regarding other factors that may be linked to the presence of comorbid ADHD, such as: an early age of onset of epilepsy [26,44], a prolonged duration of seizures, or a high frequency of seizures [27].

## 3. Diagnosis of ADHD in Children With Epilepsy

The diagnosis of ADHD is clinical, and there is currently no specific biological marker. The most commonly used diagnostic criteria are those set out in the *Diagnostic and Statistical Manual of Mental Disorders* (DSM-5) (Table 1) [45]. Although the DSM-5 diagnostic criteria remain the clinical standard for the diagnosis of ADHD, attentional and behavioural symptoms may also be interpreted in dimensional and transdiagnostic neurodevelopmental models in patients with epilepsy. This perspective recognizes the existence of shared neurobiological mechanisms between epilepsy and ADHD, as well as partially overlapping cognitive and behavioural profiles, which reinforces the need for a comprehensive and tailored clinical assessment.

**Table 1.** **ADHD symptoms and diagnostic criteria according to the DSM-5, divided into two main categories**.

DSM-5 diagnostic criteria	Description
Symptom category:	1. Often fails to give close attention to details or makes careless mistakes in schoolwork, work, or other activities.
**Inattention**	
At least 6 of the following symptoms (for children aged 16 or younger) or 5 or more (for adolescents aged 17 and over and adults). The symptoms must have persisted for at least 6 months and be inappropriate for the level of development.	2. Often has difficulty sustaining attention in tasks or play activities.
	3. Often does not seem to listen when spoken to directly.
	4. Often does not follow through on instructions and fails to finish schoolwork, chores, or duties in the workplace.
	5. Often has difficulties organizing tasks and activities.
	6. Often avoids, dislikes, or is reluctant to engage in tasks that require sustained mental effort (such as schoolwork or homework).
	7. Often loses things necessary for tasks or activities (e.g., school assignments, pencils, books, tools, keys, documents, glasses, mobile phone, etc.).
	8. Often easily distracted by extraneous stimuli.
	9. Often forgetful in daily activities.
Symptom category:	1.Often fidgets with hands or feet or squirms in seat.
**Hyperactivity/impulsivity**	2. Often runs about or climbs excessively in situations in which it is inappropriate (in adolescents or adults, this may be limited to subjective feelings of restlessness).
At least 6 of the following symptoms (for children aged 16 or younger) or 5 or more (for adolescents aged 17 and over and adults). The symptoms must have persisted for at least 6 months and be inappropriate for the level of development.	
	3. Often has difficulty playing or engaging in leisure activities quietly.
	4. Is often “on the go” or often acts as if “driven by a motor”.
	5. Often talks excessively.
	6. Often blurts out answers to questions before the questions have been completed.
	7. Often has difficulty awaiting turn.
	8. Often interrupts or intrudes on others (e.g., butts into conversations or games).
Additional Diagnostic Criteria	
• Age at onset: Some of the symptoms must be present before the age of 12.	
• Duration: The symptoms must have been present for at least 6 months.	
• Settings: The symptoms must be present in two or more settings (e.g., at home and at school or work).	
• Impact on daily life: The symptoms must interfere with social, academic or occupational functioning.	

DSM-5, Diagnostic and Statistical Manual of Mental Disorders.

This diagnostic process entails both the collection of comprehensive information from parents, carers, teachers and the patient, using a semi-structured clinical interview designed to rule out the presence of other disorders that might provide a better explanation for the symptoms presented, as well as a thorough physical and neurological examination, and an appropriate neurodevelopmental assessment (Table 2, Ref. [19,26,41,44,46,47]). This diagnosis must be performed by a clinician with training and experience in the management of ADHD and its comorbidities (a paediatric neurologist, psychiatrist or neuropsychologist) [35,46].

**Table 2.** **Specific considerations when diagnosing ADHD in people with epilepsy**.

I: Identification of comorbid conditions, including mood disorders and anxiety, sleep disorders and rational polytherapy [19,26,41,46].
II. It is essential to establish whether the ADHD symptoms were present before the onset of (comorbid) epilepsy or whether they appeared or worsened following a change in anticonvulsant medication or a worsening of the epilepsy [44].
III. In cases of seizures with different clinical features, or the acute onset or worsening of ADHD symptoms, consideration should be given to performing an EEG that includes sleep monitoring to rule out the possibility that the patient has developed epileptic encephalopathy, focusing particularly on EE-SWAS (Epileptic Encephalopathy Spike-Wave Activated in Sleep) [47].
IV. It is also sometimes necessary to distinguish between staring spells in children with predominantly inattentive ADHD and epileptic seizures (generalized absence seizures or non-motor focal seizures with partial impairment of consciousness). However, this distinction is usually possible based on the patient’s medical history, with the following factors being clinically useful: the absence of these seizures during play; if they have not been witnessed by the parents; if they can be interrupted by tactile stimuli and are not induced by hyperventilation and are more typical of a non-epileptic paroxysmal disorder; an EEG will sometimes be necessary [19,41].

Although the two conditions are closely linked, ADHD in children with epilepsy is often underdiagnosed, as the attentional and cognitive-behavioural difficulties experienced by this group are frequently attributed to the epilepsy, or to the effects of anticonvulsant medication [26].

In 2018, the Comorbidities Task Force of the *International League Against Epilepsy* (ILAE) recommended actively screening for ADHD symptoms from the age of 5–6 years, every year and following a change in anticonvulsant medication [41]. Symptoms of inattention, motor restlessness and impulsivity should also not be taken into account within 48 hours of a seizure [41].

Various authors recommend carrying out all the appropriate specific cognitive assessments in cases of poor academic performance In order to identify comorbid learning disorders [19,26,41,46].

There are as yet no specific scales for identifying symptoms associated with ADHD in people with epilepsy. In these cases, and although they are under no circumstances a substitute for a detailed clinical assessment, a structured interview with parents and children, information provided by teachers, and the judgement of the clinician making the diagnosis may be helpful for diagnosis and follow-up. The following have proven useful in this regard: the parent/teacher version of the *Child Behaviour Checklist* (CBCL) from Achenbach’s Evidence-Based Assessment System [48], the *ADHD Rating Scale* and the *Swanson, Nolan, Pelham-IV* (SNAP-IV) questionnaire, amongst others [49]. One of the scales that has proven most useful in detecting comorbidities is the Strengths and Difficulties Questionnaire (SDQ), which has been validated in Spanish and demonstrates high levels of sensitivity in identifying behavioural disorders in children aged 4–16 years, correlating with the presence of a psychiatric diagnosis, including ADHD, in 93.6% of cases [50]. Other tools that could be useful in a clinical setting for identifying impairments in executive functions are the EpiTrack Junior [51] and the Behavioural Rating Inventory of Executive Function (BRIEF) [52].

## 4. Treatment of ADHD in Children With Epilepsy

### 4.1 Non-Pharmacological Treatment

The main objectives of ADHD treatment in patients with epilepsy are to alleviate the core symptoms that characterize the condition (inattention, hyperactivity, impulsivity), improve the quality of life of the patient and their family, and prevent the onset of complications or comorbidities in the medium and long term.

The therapeutic approach to patients with epilepsy and comorbid ADHD should be similar to that adopted for patients with the same conditions but without epilepsy (multimodal treatment, including psychoeducation, psychological and behavioural interventions, educational support and pharmacotherapy) [35].

Comorbid disorders must be considered in people with epilepsy, as their treatment would improve both the symptoms of ADHD and epilepsy, such as sleep disorders and psychiatric comorbidities [53]. It is also important to consider the importance of choosing the right anticonvulsant medication; it must involve the optimization of a rational anticonvulsant therapy, while avoiding anticonvulsant medication associated with significant cognitive impairment (such as topiramate, valproic acid or GABAergic agents, such as phenobarbital and benzodiazepines) [54].

### 4.2 Pharmacological Treatment of ADHD

#### 4.2.1 Methylphenidate

Methylphenidate is a drug which acts as a stimulant of the central nervous system. Its main mechanism of action involves increasing dopaminergic and noradrenergic transmission in the prefrontal cortex and the striatum by inhibiting the dopamine transporter (DAT)1 and norepinephrine transporter (NET) presynaptic transporters respectively. It also increases the activity of the vesicular monoamine transporter (VMAT), which transports amines (e.g., dopamine, noradrenaline, serotonin and histamine) into the vesicles located in the presynaptic neurons, in preparation for their subsequent release. All these effects of methylphenidate lead to higher levels of dopamine and noradrenaline in the extracellular space. Methylphenidate is also an agonist of the 5HT1A serotonin receptors and the α2 adrenergic receptors [55,56]. It has been shown to increase histamine levels in the prefrontal cortex in animal models, and this mechanism may contribute to the regulation of attention and impulse control. However, the clinical relevance of this finding in humans has not been fully established, and most of the available evidence comes from preclinical studies [57].

Methylphenidate is available in Spain in various forms: immediate-release, in modified-release capsules containing 50:50 pellets (50% immediate-release, 50% prolonged-release) and 30:70 pellets, and in a prolonged-release form using the methylphenidate-osmotic-release oral system (OROS)® 22:78 osmotic-release system. The effective dose in children is usually between 0.5–1.5 mg/kg/day, but it should be adjusted to the individual concerned, and increased gradually (Table 3).

**Table 3.** **Main stimulant and non-stimulant medications used to treat ADHD in Spain (updated in March 2026)**.

Type of drug	Generic name	Trade name	Formats	Dosage
				(Maximum dose)
Stimulant	Methylphenidate (Immediate-release)	Rubifen®	5, 10 and 20 mg tablets	Start with 5 mg 1–2 times a day. Weekly increments of 5–10 mg.
		Medicebran®		
				(60 mg)
	Methylphenidate (50:50 modified-release)	Medikinet®	5, 10, 20, 30, 40 and 50 mg capsules	Start with 5 mg of MP IR once or twice a day, or 10 mg of Medikinet once a day. Weekly increments of 5–10 mg. Adults: Start with 10 mg, with weekly increments of 10 mg.
				(Children: 60 mg; Adults: 80 mg)
		Rubifen Prolong®	10, 20, 30, 40 and 60 mg capsules	Start with 20 mg. Weekly increments of 10–20 mg.
				(Children: 60 mg; Adults: 80 mg)
	Methylphenidate (30:70 modified-release)	Equasym®	10, 20, 30, 40 and 50 mg capsules	Start with 5 mg of MP IR once or twice a day, or 10 mg of Equasym once a day. Weekly increments of 5–10 mg.
				(Children: 60 mg; Adults: 80 mg)
	Methylphenidate (OROS® 22:78 prolonged-release)	Concerta®	18, 27, 36, 45 and 54 mg capsules	Start with 18 mg. Weekly increments of 18 mg.
		Rubicrono®		
		Atenza®		(Children: 54 mg; Adolescents and adults: 72 mg)
	Lisdexamfetamine (prolonged-release)	Elvanse®	30, 50 and 70 mg capsules	Start with 30 mg. Increments of 20 mg.
				(70 mg)
Non-stimulant	Atomoxetine	Atamax®	10, 18, 25, 40, 60 and 80 mg capsules	Children: Start with a dose of 0.5 mg/kg. Fortnightly increments up to 1.2 mg/kg/day
		Atomoxetine Cinfa®		
		Strattera®	4 mg/mL oral solution	
		Atomoxetine TecniGen®		
	Guanfacine	Intuniv®	1, 2, 3 and 4 mg tablets	Start with 1 mg per day. Weekly increments of 1 mg.
				(7 mg)
	Clonidine	Catapresan®	0.15 mg tablets	Patient ≤45 kg: start with 0.05 mg/day at bedtime. Increments every 3–7 days, and administered in divided doses every 6 hours
				(0.3 mg/day)
				Patient >45 kg: start with 0.1 mg/day at bedtime. Increments every 3–7 days, and administered in divided doses every 6 hours
				(0.4 mg/day)

The available formulations, the maximum authorized doses and the regulatory status may vary from country to country. Physicians should consult the current prescribing information and the relevant national regulatory authorizations before prescribing any of these medicines. N.B.: the doses shown are the recommendations commonly used in Spain and must be tailored to the individual patient’s age, weight, clinical response and tolerability. The approved indications, available formulations and maximum doses may vary from country to country.

#### 4.2.2 Efficacy of Methylphenidate in Patients With ADHD and Epilepsy

Both current guidelines and recent network meta-analyses based on 190 randomised clinical trials strongly support the use of psychostimulants as the most effective treatment for ADHD, taking into account both pharmacological and non-pharmacological options [35].

##### 4.2.2.1 Evidence in the Paediatric Population

The first efficacy studies of methylphenidate in children with ADHD and epilepsy focused on demonstrating improvements in various measurements of executive dysfunction, particularly planning, cognitive flexibility and inhibitory control, which is often a characteristic of children and adults with ADHD [58,59]. Several series involving 10–13 children with ADHD and epilepsy were published at the end of the last century, and reported that methylphenidate improved various executive functions as measured by different neuropsychological tests [60,61,62].

Improvements in ADHD symptoms have subsequently been demonstrated using rating scales in various studies with small sample sizes, such as the retrospective study by Socanski et al. [63] involving 14 children with ADHD and epilepsy, which reported an 85% improvement in the core symptoms of ADHD following the administration of methylphenidate, and the phase 1 trial by Gonzalez-Heydrich et al. [64], which reported an improvement in the core symptoms of ADHD in the group treated with methylphenidate-OROS® compared to the placebo group.

The efficacy of methylphenidate in the treatment of ADHD in children with epilepsy has primarily been demonstrated in two open-label studies with large sample sizes. The first study, by Gucuyener et al. [65], which assessed 57 children with epilepsy and 62 with electroencephalographic abnormalities, found that methylphenidate significantly reduced ADHD symptoms on both the Conners Scale (*p* < 0.05) and the ADHD Rating Scale (*p* < 0.001). Likewise, the results of a multi-centre observational study carried out in France by Rheims et al. [66], which included 167 children with epilepsy, of whom 46% had active epilepsy and 26% were being treated with more than two anti-epileptic drugs, showed that those who received methylphenidate experienced a significant improvement in ADHD symptoms according to the ADHD Rating Scale compared with those who did not receive psychostimulants (75% versus 42%; *p* < 0.001).

##### 4.2.2.2 Evidence in the Adult Population

Although there is less evidence in adults, the study by Adams et al. [67] conducted an open-label methylphenidate trial after a double-blind phase involving 28 adults with ADHD and epilepsy, with a mean age of 36.4 years and an average of 2.8 seizures per month, which showed an improvement in the Conners Continuous Performance Test, which assesses sustained attention in the treated group (*p* < 0.002).

In short, although whether the efficacy of methylphenidate varies according to the etiology and different epileptic syndromes is unknown, the efficacy of methylphenidate in the paediatric population with epilepsy is estimated at 65–85% [35], a similar level to that in the non-epileptic population. However, these results are based on heterogeneous populations, and the available evidence in patients with drug-resistant epilepsy and epileptic and developmental encephalopathies remains limited. The evidence in adults is more limited, although the studies available point towards the same conclusions [66,67]. Indeed, a recent Cochrane review concluded that there was sufficient evidence that methylphenidate was effective in people with ADHD and epilepsy [54]. Furthermore, the latest recommendations by the ILAE Comorbidities Task Force Comorbidities Group include a level B recommendation for the use of methylphenidate in children with epilepsy and ADHD, while atomoxetine and amphetamines were classified as Level C and level U [41].

#### 4.2.3 The Efficacy of Methylphenidate on Quality of Life and Cognitive Impairments

Patients with epilepsy frequently present a range of cognitive impairments which go beyond the executive functions and symptoms of inattention typically reported in this population. These conditions can be improved both by optimizing seizure control through rational polytherapy, and by managing the secondary effects of anticonvulsant medication, as well as by cognitive stimulation and rehabilitation [68].

Due to its multiple mechanisms of action, methylphenidate could theoretically contribute to improved cognition and memory in patients with epilepsy. In addition to the improvement brought about by its noradrenergic and dopaminergic effects, which regulate activity and communication between the ventrolateral prefrontal cortex and the hippocampal formation, methylphenidate is also thought to increase extracellular levels of histamine, which play a key role in alertness, memory and learning [57].

##### 4.2.3.1 Paediatric Population

Methylphenidate is used for the overall improvement of the cognitive dysfunction associated with conditions such as major depression and various neurodegenerative diseases [69]. However, a narrative review concluded that whilst it may improve some cognitive domains in people with ADHD, it may not improve other aspects, such as distractibility and planning [70].

However, a meta-analysis examining the cognitive effects of methylphenidate in children and adolescents with ADHD in 60 studies found statistically significant and consistent positive effects, not only on working memory but also on other aspects such as reaction time and response inhibition [71].

##### 4.2.3.2 Adult Population

Various studies involving both children and adults have also shown positive effects on memory processes, and particularly declarative and visuospatial memory [72].

These patients’ quality of life correlates positively with their ADHD symptom scores. Patients with epilepsy and ADHD score lower than patients with epilepsy and without ADHD for physical well-being, self-esteem, family and social relationships, and academic performance. Similarly, the results of a survey of 1361 adults with active epilepsy showed a poorer quality of life when ADHD symptoms were also present [73]. In this context, treatment with methylphenidate has been shown to improve quality of life in children and adults with ADHD and various types of epilepsy [35,67,74].

#### 4.2.4 Safety of Methylphenidate in Patients With Epilepsy

Although psychostimulants, and amphetamine derivatives in particular, were initially considered potential anticonvulsant medications, based on the theory that dopamine (DA) receptor signalling may suppress seizure activity, the properties of methylphenidate as a CNS stimulant raised concerns amongst clinicians, given the possibility that it might increase the risk of seizures by lowering the seizure threshold [60]. In fact, the information in various prescribing documents states that methylphenidate “should be used with caution in patients with epilepsy” and US and European regulatory agencies warn that “stimulants may lower the seizure threshold and should be discontinued if the frequency of seizures increases”.

##### 4.2.4.1 Safety in the Paediatric Population

This concern increased following reports of isolated cases involving children without epilepsy, in which the beginning of methylphenidate treatment, in combination with sertraline in many cases, was linked to the onset of seizures [75]. Hemmer et al. [76] highlighted this potential risk of increased epileptiform activity in a study in which serial EEGs were performed on a group of 234 children without epilepsy, in which they concluded that methylphenidate could trigger seizures in children without epilepsy (an incidence of 0.6%); particularly in cases with a history of interictal epileptic activity (an incidence of 10%). This risk has not been confirmed in two subsequent large-scale observational studies. Neither a study carried out in Hong Kong, which involved 29,604 children and young adults diagnosed with ADHD who were taking methylphenidate [77], or a study carried out in the United States, in which the population monitored amounted to more than 800,000 people, found that treatment with methylphenidate led to an increased risk of seizures [4]. This concern was greater in patients with epilepsy.

A single prospective study involving a small sample of 33 children with ADHD and epilepsy, which compared the frequency of seizures between those who started treatment with methylphenidate-OROS *and those* on placebo, reported an increased risk of seizures, which was directly proportional to the dose of methylphenidate used: 0.53 seizures/100 days in the placebo group, 1.12 seizures/100 days in those who took 36 mg, and 2.30 seizures/100 days in the 54 mg group (*p* < 0.001). However, the authors pointed out that these findings would need to be verified in further studies due to the significant limitations of their study: the small and heterogeneous sample, and the low baseline seizure frequency (the inclusion criterion was having had a seizure in the previous 5 years) [64].

The main limitations of all these studies are that the sample sizes were small, other risk factors for seizures were not analysed, and most of the authors concluded that further studies would be needed to confirm this link between methylphenidate and seizures, as their studies did not provide sufficient evidence of causality [60,64,74].

Nine Class II studies and 10 Class III studies have since been published, involving between 11 and 167 patients, which have found no evidence that methylphenidate may cause an increase in the frequency or severity of seizures, even in children [64,65,66,74].

##### 4.2.4.2 Safety in the Adult Population

Park et al. [78] published a retrospective study of 105 adolescents and young adults with epilepsy, in which they reported that the use of methylphenidate approximately 20% of participants experienced seizure aggravation. These findings were confirmed in various subsequent studies involving adult populations [67,74,79].

##### 4.2.4.3 Safety in Drug-Resistant Epilepsy

Most studies showed that treatment with methylphenidate did not increase the risk of seizures in patients with refractory epilepsy. However, there are two studies with a small sample size that suggest a degree of caution is warranted. In the first, Koneski et al. [80] reported an increase in the frequency of seizures at 6 months in 2 out of 24 patients, but a reduction in the severity of seizures in the sample as a whole. Subsequently, in a similar study, Santos et al. [81] reported that 4 out of 22 children with refractory epilepsy experienced an increase in seizure frequency three months after starting treatment with methylphenidate.

##### 4.2.4.4 Safety Conclusions

A number of population-based studies have been published in recent years, which have provided additional important information about the safety of methylphenidate and people with epilepsy. According to the Swedish National Prescribed Drug Register for 2006–2013, 995 paediatric patients with epilepsy experienced a reduction in seizure frequency in the first 24 weeks following the start of pharmacological treatment for ADHD (HR 0.73; 95% CI: 0.57–0.94) [3]. Likewise, a retrospective study in the USA (undertaken between 1999 and 2010), which compared a group of 18,166 children with epilepsy and ADHD who had begun treatment with stimulants with a group of 54,197 children with ADHD who were not receiving psychostimulant treatment, found no higher rate of hospitalization related to epileptic seizures (HR 0.95; 95% CI: 0.83–1.09 and HR 0.99; 95% CI: 0.85–1.15) [1]. Furthermore, observational studies found that the dose of methylphenidate or the etiology was associated with an increased risk of epileptic seizures. As a result, both the latest Cochrane reviews and the recommendations of the ILAE Comorbidities Task Force consider methylphenidate to be safe, with a level B recommendation [41,54].

The evidence currently available does not permit firm conclusions to be drawn about the possible differences in risk depending on the epilepsy syndrome, etiology or the degree of seizure control. Although methylphenidate appears to be safe in most patients with epilepsy, closer and more tailored monitoring is recommended in those with drug-resistant epilepsy, a recent worsening of seizures, or epileptic and developmental encephalopathies. In these patients, the use of methylphenidate must be tailored to the individual following a careful assessment of the benefit-risk balance and close clinical monitoring.

Given that most patients with epilepsy experience spontaneous fluctuations in their seizure frequency, the onset of a worsening of symptoms following the start of methylphenidate treatment does not necessarily imply a causal relationship. Other potentially contributory factors must be ruled out before attributing this deterioration to the drug. The authors propose a management algorithm for cases in which the deterioration of symptoms is suspected to be due to methylphenidate (Fig. 2).

**Fig. 2.**
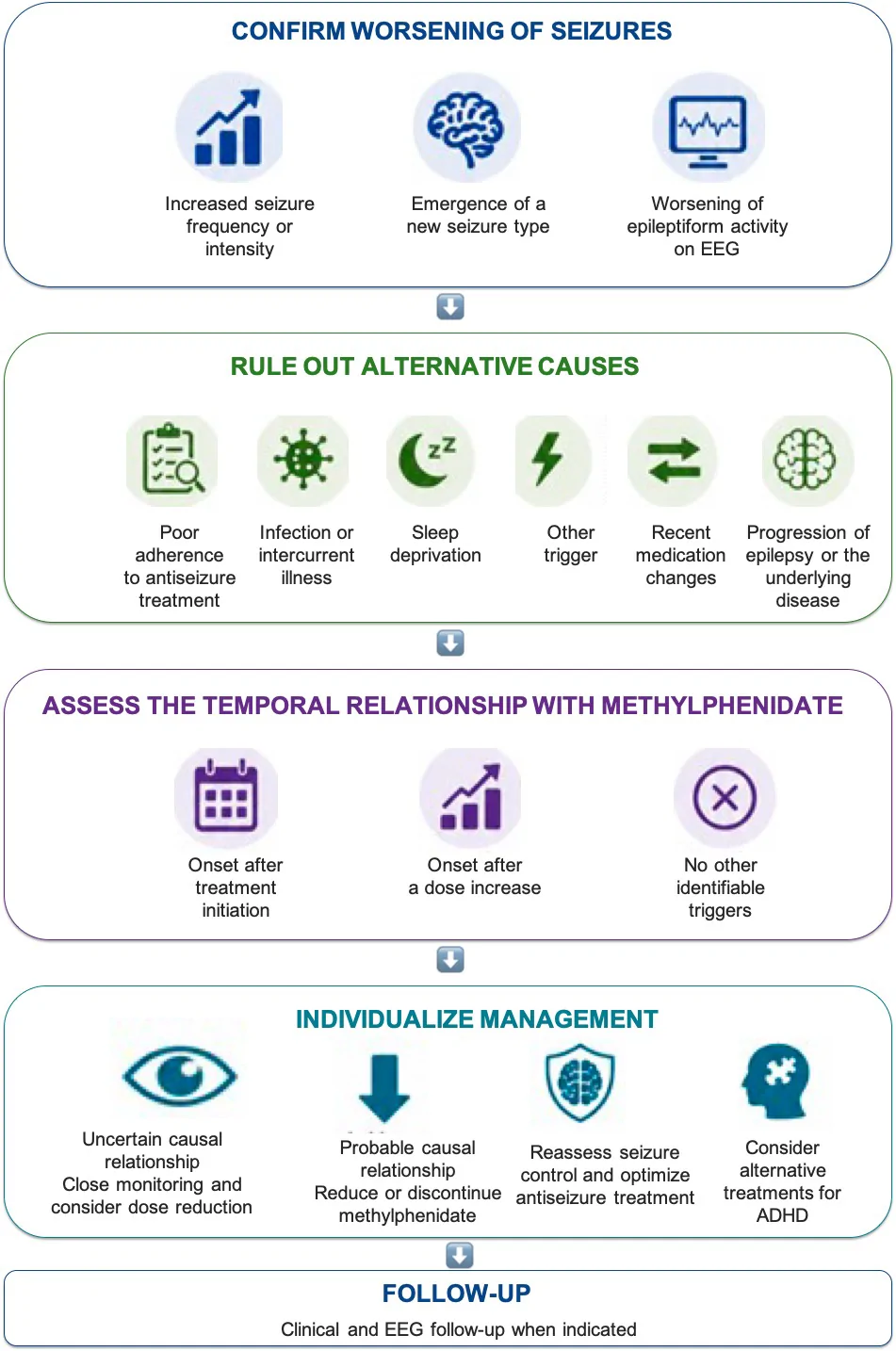
**Recommendations in cases with a suspected worsening of epilepsy due to methylphenidate**.

Other potentially relevant adverse effects must be taken into account when assessing the safety of methylphenidate in patients with epilepsy. These include a loss of appetite, weight loss, insomnia and other sleep disorders. The latter is particularly important in patients with epilepsy, as sleep deprivation can increase the likelihood of seizures. Methylphenidate has a relatively low potential for clinically relevant pharmacokinetic interactions with most anti-seizure medicines. However, due to the frequent polytherapy in patients with epilepsy, tailored monitoring is recommended following the beginning or modification of treatment.

## 5. Limitations

This review has a number of limitations that should be taken into account when interpreting its recommendations. First, it is a narrative review of the literature rather than a systematic review, and although an extensive search was carried out for the available scientific evidence, no formal methodology for the selection and evaluation of studies based on Preferred Reporting Items for Systematic Reviews and Meta-Analyses (PRISMA) criteria was followed. The proposed recommendations should therefore be interpreted as practical guidance based on the best available scientific evidence and the consensus of the experts, rather than as recommendations based on a systematic review with a formal assessment of the quality of the evidence.

Second, the proposed recommendations are based on both published evidence and the consensus of experts. Although the panel consisted of paediatric neurologists from five Spanish hospitals with extensive experience in epilepsy and neurodevelopmental disorders, neither a formal Delphi methodology nor a structured voting system was used to quantify the level of consensus reached.

The quality of the available evidence also remains limited. Most of the published studies on the use of methylphenidate in patients with epilepsy and ADHD are observational, retrospective or have small sample sizes. There have been few randomised controlled clinical trials specifically designed to assess the efficacy and safety of this treatment in this population. Furthermore, the available evidence does not offer firm conclusions regarding possible differences in safety based on epileptic syndrome, etiology or the degree of seizure control.

Another major limitation is the heterogeneity of the patients included in the studies available, encompassing different ages, types of epilepsy, etiologies, degrees of seizure control and concomitant anti-seizure treatments, making it difficult to extrapolate the results to specific subgroups of patients.

Finally, although data from both paediatric and adult populations have been included to provide a more comprehensive overview of the available evidence, most of the studies involved children and adolescents. Consequently, some recommendations applicable to adults are based on a smaller body of evidence, and should be interpreted with caution until specific studies of higher methodological quality become available.

## 6. Recommendations of the Group of Experts and Conclusion

Despite long-standing concerns about the safety of stimulant medication in patients with epilepsy, evidence of an increased risk of seizures associated with the use of ADHD medication is limited and based mainly on case series reports, while large cohorts and population-based registries suggest that methylphenidate does not increase the incidence of seizures, but may even reduce the risk of them occurring [41,54].

As ADHD has a significant adverse impact on the academic, social and cognitive functioning of patients with epilepsy and their families, its treatment is of crucial importance.

The authors of this review acknowledge that randomized, placebo-controlled trials are needed to enhance the evidence available regarding the efficacy and particularly the safety of methylphenidate in people with epilepsy and ADHD [53]. The available evidence is particularly strong in the paediatric population, whilst data for adults are more limited, but generally consistent regarding the efficacy and safety of methylphenidate.

Despite the need to improve the evidence available regarding the pharmacological treatment of ADHD in children and adults with epilepsy, any comprehensive management of patients with epilepsy must include the treatment of their comorbid conditions, including ADHD. While methylphenidate is a drug that has been shown to be effective in treating ADHD symptoms in children and adults with epilepsy (to a similar extent as in the population without epilepsy), the available evidence does not suggest that the majority of patients with epilepsy treated with methylphenidate experience a clinically relevant increase in the frequency of epileptic seizures, although an increased risk in certain populations for which the available evidence remains limited cannot be entirely ruled out.

For all these reasons, this group of experts believes that methylphenidate may be a therapeutic option for children and adults with epilepsy and ADHD when clinically indicated, following an appropriate diagnostic assessment and the optimization of treatment for epilepsy, its comorbidities and non-pharmacological interventions. The decision to start treatment must be made on a case-by-case basis, particularly in patients with active epilepsy, drug-resistant epilepsy, a recent worsening of seizures, or epileptic and developmental encephalopathies (Fig. 1).
