# Supplementary material for: Expert Review on the Use of Methylphenidate in Children and Adult With Epilepsy and Attention Deficit Hyperactivity Disorder
Source: Rev Neurol. 2026 Oct 29;81(10):52291. doi: 10.31083/RN52291 (PMC13732423; doi:10.31083/RN52291)
Supplement: Translation

# Translation: Revisión de Expertos Sobre el Uso de Metilfenidato en Niños y en adultos con Epilepsia y Trastorno por Déficit de Atención e Hiperactividad

**Authors:** Víctor Soto Insuga, María Eugenia Russi, Elena González Alguacil, Javier López Pisón, Sergio Aguilera Albesa, Ignacio Málaga Dieguez, Carmen Fons

**Affiliations:** ^1^Unidad de Neurología, Hospital Niño Jesús, 28009 Madrid, España; ^2^Área de Neurología Pediátrica y Neurofisiología. Hospital Sant Joan de Déu. Institut de Recerca Sant Joan de Déu. EpiCARE Coordinator Center, 08950 Barcelona, España; ^3^Servicio de Pediatría, Hospital Miguel Servet, 50009 Zaragoza, España; ^4^Unidad de Neuropediatría, Servicio de Pediatría, Hospital Universitario de Navarra, 31008 Pamplona, España; ^5^Unidad de Neurología Pediátrica, AGC de Pediatría, Hospital Universitario Central de Asturias, 33011 Oviedo, España

**Keywords:** epilepsia, metilfenidato, neurología, pediatría, trastorno por déficit de atención e hiperactividad (TDAH)

## Abstract

El trastorno por déficit de atención e hiperactividad (TDAH) es una comorbilidad que puede estar presente hasta en el 12%-39% de la población con epilepsia, produciendo un impacto significativo tanto a nivel cognitivo-conductual como en el área psicosocial y en la calidad de vida. Además, el TDAH y la epilepsia presentan una relación bidireccional. A pesar de que el metilfenidato es un tratamiento que ha demostrado ser altamente eficaz en la mejora de los síntomas nucleares del TDAH, sigue estando actualmente infrautilizado en las personas con epilepsia debido a la creencia previa de hace más de 20 años de que el uso de psicoestimulantes pudiera aumentar la frecuencia de crisis. El presente trabajo consiste en una revisión narrativa de la literatura científica publicada entre enero de 2000 y mayo de 2026 sobre el uso de metilfenidato en pacientes con epilepsia y TDAH. A partir de la evidencia identificada, un grupo de neuropediatras de cinco hospitales españoles con experiencia en epilepsia y trastornos del neurodesarrollo elaboró, mediante consenso de expertos, una serie de recomendaciones prácticas para la evaluación y el tratamiento de estos pacientes. Entre ellas destacan la búsqueda activa de los síntomas de TDAH en todos los niños y adultos con epilepsia, desde su debut. La evidencia actual no ha demostrado de forma consistente un aumento del riesgo de crisis asociado al uso de metilfenidato en pacientes con epilepsia, aunque los datos disponibles siguen siendo limitados para algunos subgrupos específicos de epilepsia. Asimismo, el infratratamiento del TDAH supone un empeoramiento del pronóstico de estos pacientes. El metilfenidato puede considerarse en niños y adultos con epilepsia cuando exista una indicación clínica para el tratamiento del TDAH, una vez optimizado el manejo de la epilepsia, sus comorbilidades y las intervenciones conductuales y psicosociales. La decisión terapéutica debe individualizarse, especialmente en pacientes con epilepsia activa, epilepsia farmacorresistente, empeoramiento reciente de las crisis o encefalopatías epilépticas y del desarrollo.

## 1. Introducción

El trastorno por déficit de atención e hiperactividad (TDAH) es considerado hoy en día como uno de los trastornos del neurodesarrollo más frecuentes y motivo de consulta habitual en la práctica clínica del neuropediatra. El TDAH es un trastorno de origen neurobiológico caracterizado por dificultades en la regulación del nivel de actividad (hiperactividad), la inhibición de los pensamientos y/o comportamientos (impulsividad) y la capacidad de mantener la atención en tareas que requieren un esfuerzo cognitivo (inatención) [1,2,3,4,5]. Se estima una prevalencia mundial en torno al 5–7% en la infancia y en 1–5% en la población adulta [6,7]; y de un 6,8% en la población infantil española [8].

Por otro lado, la epilepsia se define como un trastorno cerebral caracterizado por una predisposición continuada a la generación de crisis epilépticas que se asocia con consecuencias neurobiológicas, cognitivo-conductuales y sociales constituyendo un motivo de consulta altamente frecuente en neuropediatría. Su prevalencia en Europa es del 0,7% de la población, afectando a unos seis millones de habitantes (entre 300.000 y 400.000 en España), con 400.000 nuevos casos cada año y una tasa de incidencia mundial de 61,44 × 100.000 personas/año [9]. Los niños con epilepsia presentan una alta tasa de comorbilidades psiquiátricas y neurológicas (trastornos del aprendizaje, trastornos del estado de ánimo, ansiedad, trastorno del espectro autista (TEA) y alteraciones cognitivas, entre otros). Entre todas estas condiciones, destaca el TDAH, que representa la comorbilidad más frecuentemente asociada en niños con epilepsia [10].

Según varios metaanálisis recientes, en niños, la presencia de TDAH previo al inicio de la epilepsia es frecuente, estimándose una prevalencia de 12–39%, lo que supone un riesgo entre 3 y 5 veces mayor que en población sin epilepsia [11,12,13,14,15,16,17,18]. Por otra parte, en niños con epilepsia farmacorresistente, la prevalencia de TDAH es aún mayor, alcanzándose hasta en el 70% de los casos [19]. Esta relación, sin embargo, es bidireccional [20], de forma que la epilepsia también puede estar presente en el 3,4% de las personas con TDAH (2,53–4,21%) [11,21]. Se estima que hasta el 20–50% de los niños con epilepsia presentan síntomas subclínicos de TDAH [22]. Esta asociación altamente frecuente es debida a que tanto la epilepsia como el TDAH presentan mecanismos neurobiológicos compartidos entre los que se incluyen redes neuronales aberrantes fronto-cerebelo-estriatales, alteración en la funcionalidad de canales iónicos, desequilibrios en la neurotransmisión [23], relacionadas con factores genéticos, con la propia actividad epileptiforme, y epigenéticos, que afectan la plasticidad y regeneración neuronal y que podrían contribuir tanto a la hiperexcitabilidad neuronal característica de la epilepsia como a las alteraciones atencionales y disejecutivas observadas en el TDAH [11,18]. En este sentido, determinadas enfermedades genéticas del neurodesarrollo presentan una prevalencia elevada de ambas entidades. Entre ellas destacan las distrofinopatías, en las que se ha descrito una mayor frecuencia tanto de epilepsia como de síntomas de TDAH y otras alteraciones cognitivas y conductuales, así como el síndrome de Noonan y otras rasopatías, en las que las alteraciones de las vías de señalización implicadas en el desarrollo cerebral podrían contribuir a la aparición de trastornos del neurodesarrollo y epilepsia [24].

Si bien en lo que a las características propias del TDAH en niños con epilepsia no parece existir una diferencia significativa respecto a la población sin epilepsia [25], algunos autores refieren una mayor proporción del subtipo inatento en casos de epilepsia comórbida [26,27]. Por otra parte, mientras que el TDAH afecta predominantemente al sexo masculino en relación al femenino, con una proporción de 2:4–4:1 (según sean estudios clínicos u observacionales), en niños con epilepsia, esta diferencia según sexo no existe [28,29,30].

### Justificación de Elaboración de Recomendaciones de Expertos

Además de ser la comorbilidad más frecuentemente reportada en niños con epilepsia, el TDAH impacta negativamente en diferentes áreas del individuo, que abarcan desde los aprendizajes y el rendimiento académico, hasta aspectos relacionados con la cognición social, las funciones ejecutivas, el estado de ánimo y la ansiedad. Esto representa uno de los factores más importantes que condicionan una peor calidad de vida tanto en el niño con epilepsia como en sus familiares [31,32]. Asimismo, los síntomas de inatención, inquietud motriz e impulsividad suelen persistir en la vida adulta del paciente con epilepsia, con una persistencia sintomática estimada del 15–18% de los adultos con esta condición, y una prevalencia estimada de TDAH de más del doble que la de personas sin epilepsia. Por todo ello, dentro del abordaje holístico del paciente con epilepsia, resulta de fundamental importancia el tratamiento específico del TDAH comórbido [33,34].

Respecto al tratamiento, existe desde hace años consenso acerca de que el abordaje terapéutico de todo paciente con TDAH debe ser multimodal; es decir, que incluya la combinación de intervenciones psicopedagógicas, de reeducación y apoyo escolar, junto con el tratamiento psicológico cognitivo-conductual, de psicoeducación, apoyo psico-social en el entorno y el tratamiento farmacológico a partir de los 6 años [35]. En lo que al tipo de tratamiento farmacológico respecta, numerosos estudios controlados han demostrado la eficacia tanto de los fármacos psicoestimulantes como de los no-psicoestimulantes. Sin embargo, actualmente existe consenso en que los fármacos de primera elección para el tratamiento del TDAH son los estimulantes del sistema nervioso central (SNC) o psicoestimulantes, como el metilfenidato y la lisdexanfetamina. Estos medicamentos presentan un mayor tamaño de efecto y un menor número necesario a tratar en comparación con los no psicoestimulantes; situando a los estimulantes entre los fármacos más empleados por su eficacia y seguridad, en el tratamiento del TDAH. Dentro de este grupo farmacológico, el metilfenidato es considerado en nuestro entorno como el fármaco de primera elección en el tratamiento del TDAH, con una eficacia estimada alrededor del 65%–85% [35].

A pesar de la importancia de identificar precozmente el TDAH en pacientes con epilepsia, es frecuente que esté infradiagnosticado y que, por tanto, no reciban un tratamiento farmacológico adecuado [26]. Esta situación se debe, en parte, a una preocupación histórica sobre la seguridad del uso de psicoestimulantes en pacientes con epilepsia, ya que se suponía que podían reducir el umbral de crisis y aumentar la morbilidad relacionada. Sin embargo, estudios recientes han demostrado que los psicoestimulantes, como el metilfenidato, pueden utilizarse de manera segura sin incrementar la frecuencia de las crisis, en pacientes con epilepsia. Dado que actualmente no existe un tratamiento específico para el TDAH asociado a la epilepsia, es fundamental revisar la evidencia científica disponible hasta la fecha y elaborar recomendaciones basadas en la evidencia y en la opinión de expertos.

Con el objetivo de abordar esta problemática, un grupo de neuropediatras de cinco hospitales españoles (especializados en epilepsia y trastornos del neurodesarrollo, con especial atención al TDAH en niños y adolescentes), hemos realizado una revisión de la evidencia publicada desde enero de 2000 hasta mayo de 2026, analizando más de 140 artículos. Posteriormente, se estableció una serie de recomendaciones que se recogen en la Fig. 1.

**Fig. 1.** **Recomendaciones del grupo de expertos para el manejo de pacientes con TDAH y epilepsia**. TDAH, trastorno por déficit de atención e hiperactividad; EEG, electroencefalograma.

El presente trabajo consiste en una revisión narrativa de la literatura científica publicada entre enero de 2000 y mayo de 2026 sobre el uso de metilfenidato en pacientes con epilepsia y TDAH, complementada con la elaboración de recomendaciones prácticas basadas en el consenso de un grupo de expertos. La búsqueda bibliográfica se llevó a cabo principalmente en PubMed/MEDLINE, complementándose con la revisión manual de las referencias de los artículos identificados y de documentos relevantes publicados por sociedades científicas internacionales. Se utilizaron diferentes combinaciones de términos relacionados con “epilepsy”, “attention deficit hyperactivity disorder”, “ADHD”, “methylphenidate”, “stimulants”, “psychostimulants”, “children” y “pediatric”. Esta estrategia de búsqueda permitió identificar más de 140 artículos relevantes seleccionados por su relevancia clínica y científica para los objetivos del documento, incluyendo estudios observacionales, ensayos clínicos, revisiones sistemáticas, metaanálisis, documentos de consenso y guías de práctica clínica. Dado que el objetivo de este trabajo fue elaborar una revisión narrativa con recomendaciones prácticas basadas en la experiencia clínica, no se aplicó una metodología propia de una revisión sistemática. En consecuencia, no se realizó una evaluación formal del riesgo de sesgo de los estudios incluidos, ni una graduación de la calidad de la evidencia mediante sistemas estandarizados (como *Grading of Recommendations Assessment, Development and Evaluation* (GRADE)), ni un metaanálisis cuantitativo. La selección de los artículos se realizó en función de su relevancia clínica y científica, complementada por el juicio experto del grupo de trabajo.

Tras la revisión de la literatura científica identificada, se constituyó un panel de expertos integrado por neuropediatras de cinco hospitales españoles con experiencia en epilepsia y trastornos del neurodesarrollo. El proceso de consenso incluyó tres reuniones telemáticas en las que se revisó y discutió la evidencia disponible. A partir de dicha revisión, se elaboraron propuestas de recomendaciones prácticas que fueron debatidas, modificadas y refinadas de forma iterativa hasta alcanzar un acuerdo entre todos los participantes. Las recomendaciones finales reflejan el consenso del grupo de expertos basado tanto en la evidencia científica disponible como en la experiencia clínica acumulada de los autores que se recogen en la Fig. 1.

## 2. Factores de Riesgo y Comorbilidades del TDAH en Población con Epilepsia 

Existen varios factores de riesgo que se han relacionado de forma estadísticamente significativa con el desarrollo del TDAH en pacientes con epilepsia, así como determinados síntomas comórbidos frecuentes que pueden agravar los síntomas propios del TDAH y que, por tanto, es fundamental poder identificar. Los trastornos cognitivos, los antecedentes familiares de TDAH y de epilepsia, la refractariedad de las crisis, las anomalías epileptiformes, el tipo de epilepsia, los medicamentos anti-crisis (MACs), los trastornos psiquiátricos y los trastornos del sueño suponen aspectos fundamentales a tener en cuenta para garantizar un adecuado manejo de este tipo de pacientes.

### 2.1 Trastornos Cognitivos

La presencia de discapacidad intelectual se identifica como un factor de riesgo independiente para la presencia de TDAH en niños con epilepsia [27]. Por tanto, en aquellos niños con epilepsia y discapacidad intelectual, el riesgo de presentar un TDAH asociado es mayor. En un estudio en 85 niños con epilepsia, el TDAH estaba presente en el 20% de aquellos pacientes con un nivel cognitivo normal en relación al 59% de aquellos con discapacidad intelectual [36].

### 2.2 Antecedentes Familiares de TDAH y de Epilepsia

Se calcula que la tasa de heredabilidad de TDAH global es del 76% [27]. En población con epilepsia, sin embargo, existe menor evidencia al respecto, destacando un pequeño estudio en 16 niños con TDAH y epilepsia, en el que se demostró que el 50% de las madres cumplían criterios de TDAH [37]. En una reciente revisión de 46 estudios, la presencia de familiares con epilepsia suponía un riesgo 2,58 mayor de desarrollar TDAH [27].

### 2.3 Refractariedad y Tipo de Epilepsia

Aunque el TDAH suele ser más prevalente en epilepsias refractarias [38], también es una comorbilidad altamente frecuente en epilepsias autolimitadas y que no suelen requerir muchas líneas de tratamiento anticrisis. Entre ellas se incluye la epilepsia con puntas centrotemporales (SeLECTS, del inglés *Self-limited Epilepsy with Centro-Temporal Spikes*), en la que el TDAH suele estar presente en más de la mitad de los casos (se estima que en un 60%) [39], o la epilepsia de tipo ausencia infantil, en las que el porcentaje de TDAH es de hasta el 26–57% [27,40].

Si bien el TDAH puede estar presente en epilepsias de diferente localización, parece ser más frecuente en aquellas de localización frontal debido a que el córtex prefrontal y el giro frontal inferior resultan ser áreas clave para el desarrollo adecuado de las funciones ejecutivas [28].

### 2.4 Medicamentos Anti-Crisis

Si bien cualquier MAC con potencial afectación cognitiva (especialmente los GABAérgicos), debería considerarse como factor potenciador de la exacerbación de la sintomatología TDAH. El único MAC en el que se ha demostrado un nivel de evidencia Grado A que podría empeorar los síntomas de inatención, es el ácido valproico, llevando a interrumpir el tratamiento hasta en el 25% de los casos [41]. Asimismo, diferentes revisiones demuestran que la politerapia se relaciona con mayor probabilidad de desarrollar TDAH [27].

Los datos actuales sugieren que fármacos de nueva generación como brivaracetam, perampanel o cenobamato presentan, en general, un perfil cognitivo relativamente neutro. Por otra parte, algunos tratamientos recientemente incorporados al manejo de determinadas encefalopatías epilépticas y del desarrollo, como cannabidiol y fenfluramina, han mostrado efectos favorables sobre aspectos relacionados con el estado de alerta, la interacción social y conducta más allá de su efecto anticrisis. No obstante, la evidencia disponible es aún insuficiente para establecer recomendaciones específicas respecto a su posible impacto sobre los síntomas nucleares del TDAH [42].

### 2.5 Trastornos Psiquiátricos

Los trastornos del estado de ánimo, la ansiedad, el trastorno de conducta y los trastornos conductuales (entre los que se encuentran el trastorno oposicionista desafiante) son altamente frecuentes, tanto en población con epilepsia como en niños con TDAH, por lo que se recomienda identificar de forma precoz [41].

### 2.6 Trastornos de Sueño

Se estima que hasta el 84% de los niños con epilepsia presentan algún trastorno del sueño, entre los que destacan: insomnio (13%–31%), hipersomnia (10%–47%), parasomnias (52%) y síndrome de apnea del sueño (10%–30%). Igualmente, el 25–70% de los niños con TDAH presentan frecuentes trastornos de sueño [43].

### 2.7 Anomalías Epileptiformes

La presencia de anomalías epileptiformes intercríticas es frecuente en niños con epilepsia y TDAH, estimándose que hasta el 51% presentan anomalías epileptiformes intercríticas al realizar un electroencefalograma (EEG) de sueño, comparado con una incidencia de 1,4%–6,5% de anomalías epileptiformes en niños sin epilepsia [41]. Sin embargo, son varios los estudios que concluyen que no existe en el momento actual, evidencia de una relación estadísticamente significativa entre cambios EEG interictales y la gravedad del déficit de atención [27].

### 2.8 Otros Factores

No existe aún consenso o los estudios son contradictorios sobre otros factores que podrían estar en relación con la presencia de un TDAH comórbido, como son: la edad temprana de debut de la epilepsia [26,44], la duración prolongada de las crisis, o la alta frecuencia de crisis [27].

## 3. Diagnóstico de TDAH en Niños con Epilepsia

El diagnóstico de TDAH es clínico y no existe al momento actual un marcador biológico específico, siendo los criterios diagnósticos más utilizados los establecidos en el manual de psiquiatría infantil DSM-5 (del inglés *Diagnostic and Statistical Manual of Mental Disorders*) (Tabla 1) [45]. Aunque los criterios diagnósticos del DSM-5 continúan constituyendo el estándar clínico para el diagnóstico del TDAH, en los pacientes con epilepsia los síntomas atencionales y conductuales también pueden interpretarse dentro de modelos dimensionales y transdiagnósticos del neurodesarrollo. Esta perspectiva reconoce la existencia de mecanismos neurobiológicos compartidos entre la epilepsia y el TDAH, así como perfiles cognitivos y conductuales parcialmente solapados, lo que refuerza la necesidad de una valoración clínica integral e individualizada.

**Tabla 1.** **Síntomas y criterios diagnósticos del TDAH según el DSM-5, divididos en las dos principales categorías**.

Criterios diagnósticos del DSM-5	Descripción
Categoría de síntomas:	1. A menudo falla en prestar atención a los detalles o comete errores por descuido en tareas escolares, laborales o durante otras actividades.
**Falta de atención**	
Al menos 6 o más de los siguientes síntomas (para niños menores de 16 años o menos) o 5 o más (para adolescentes de 17 años en adelante y adultos). Los síntomas deben haber persistido durante al menos 6 meses y ser inapropiados para el nivel de desarrollo.	
	2. A menudo tiene dificultades para mantener la atención en tareas o actividades recreativas.
	3. A menudo parece no escuchar cuando se le habla directamente.
	4. A menudo no sigue instrucciones y no termina tareas escolares, quehaceres o responsabilidades laborales.
	5. A menudo tiene dificultades para organizar tareas y actividades.
	6. A menudo evita, le disgusta o es reacio a participar en tareas que requieren un esfuerzo mental sostenido (como tareas escolares o tareas domésticas).
	7. A menudo pierde cosas necesarias para tareas y actividades (materiales escolares, lápices, libros, herramientas, llaves, documentos, anteojos, teléfonos móviles, etc.).
	8. A menudo se distrae fácilmente con estímulos externos
	9. A menudo es olvidadizo en las actividades diarias.
Categoría de síntomas:	1. A menudo se mueve inquieto o retuerce las manos o los pies, o se remueve en su asiento.
**Hiperactividad/impulsividad**	
Al menos 6 o más de los siguientes síntomas (para niños menores de 16 años o menos) o 5 o más (para adolescentes de 17 años en adelante y adultos). Los síntomas deben haber persistido durante al menos 6 meses y ser inapropiados para el nivel de desarrollo.	2. A menudo corretea o trepa en situaciones inapropiadas. (en adolescentes o adultos, puede limitarse a sentir inquietud “interna”).
	3. A menudo es incapaz de jugar o participar en actividades tranquilas.
	4. A menudo está “en movimiento” o actúa como si “tuviera un motor”.
	5. A menudo habla excesivamente.
	6. A menudo responde bruscamente antes de que se complete una pregunta.
	7. A menudo tiene dificultades para esperar su turno.
	8. A menudo interrumpe o se entromete en conversaciones o juegos de otros.
Criterios Adicionales Para el Diagnóstico	
• Edad de aparición: Algunos de los síntomas deben estar presentes antes de los 12 años.	
• Duración: Los síntomas deben estar presentes por al menos 6 meses.	
• Entornos: Los síntomas deben estar presentes en dos o más contextos (por ejemplo, en casa y en la escuela o trabajo).	
• Impacto en la vida diaria: Los síntomas deben interferir en el funcionamiento social, académico o laboral.	

DSM-5, Diagnostic and Statistical Manual of Mental Disorders.

En este proceso diagnóstico se requiere tanto de una recogida amplia de información proveniente de padres, cuidadores, profesores y del propio afectado mediante la realización de una entrevista clínica semiestructurada que permita excluir la presencia de otros trastornos que pudieran explicar mejor la sintomatología presentada, así como de una exploración física y neurológica completa, y una adecuada valoración del neurodesarrollo (Tabla 2, Ref. [19,26,41,44,46,47]). Este diagnóstico debe realizarlo cualquier clínico con formación y experiencia en manejo del TDAH y sus comorbilidades (neuropediatra, psiquiatra o neuropsicólogo) [35,46].

**Tabla 2.** **Aspectos específicos a tener en cuenta en el diagnóstico de TDAH en población con epilepsia**.

I: Una identificación de comorbilidades, cuyo tratamiento puede suponer una mejora de los síntomas de TDAH, que incluyen las alteraciones del estado de ánimo-ansiedad, alteraciones de sueño y una politerapia racional entre otros [19,26,41,46].
II. Será fundamental conocer si los síntomas de TDAH estaban presentes antes del debut de epilepsia (comórbida) o aparecieron/empeoraron tras algún cambio de MAC o empeoramiento de la epilepsia [44].
III. En caso de aparición de crisis de diferente semiología o aparición/empeoramiento agudo de sintomatología de TDAH debería plantearse la posibilidad de realizar EEG que incluya sueño para descartar la posibilidad de que haya desarrollado una encefalopatía epiléptica con especial atención a EE-SWAS (del inglés Epileptic Encephalopathy Spike-Wave Activated in Sleep) [47].
IV. También en ocasiones será fundamental diferenciar la presencia de ensimismamientos presentes en los niños con TDAH de predominio inatento versus crisis epilépticas (generalizadas tipo ausencia o focales no-motoras con alteración parcial de consciencia). Aunque con una anamnesis se suelen diferenciar, siendo de ayuda clínica: ausencia de estos durante el juego, no haberlas presenciados por los padres, que se puedan interrumpir por estímulos táctiles y no inducidas por hiperventilación más características de un trastorno paroxístico no epiléptico; en ocasiones será necesario la realización de un EEG [19,41].

A pesar de estar íntimamente relacionados, el TDAH en niños con epilepsia suele estar infradiagnosticado, debido a que las dificultades atencionales y cognitivo-conductuales de esta población, suelen atribuirse a la propia epilepsia o a los efectos de los MAC [26].

Desde el Grupo de Trabajo de Comorbilidades de la ILAE (*International League Against Epilepsy*) en 2018 se recomienda realizar una búsqueda activa de síntomas TDAH desde los 5–6 años de forma anual y tras el cambio de MAC [41]. Igualmente se recomienda no considerar los síntomas de inatención, inquietud motriz e impulsividad en las 48 horas tras una crisis [41].

Para la detección de trastornos de aprendizaje comórbidos, diferentes autores recomiendan llevar a cabo todas aquellas valoraciones cognitivas específicas que sea oportunas, en caso de malos resultados escolares [19,26,41,46].

No existen hasta el presente, escalas específicas para la detección de los síntomas asociados del TDAH en población con epilepsia. En estos casos, pueden ser de ayuda para el diagnóstico y el seguimiento, aunque en ningún caso pueden ni deben sustituir la evaluación clínica detallada, la entrevista estructurada a padres y niños, la información aportada por los profesores, y el criterio del clínico que realiza el diagnóstico. En este sentido han demostrado su utilidad la versión para padres/profesores del *Child Behaviour Checklist* (CBCL) del Sistema de Evaluación Basada en la Evidencia de Achenbach [48], la *ADHD Rating Scale* y el cuestionario *Swanson, Nolan, Pelham-IV* (SNAP-IV) entre otras [49]. Una de las escalas que más utilidad ha demostrado en la detección de comorbilidades, es el Cuestionario de Cualidades y Dificultades (SDQ, del inglés *Strengths and Difficulties Questionnaire*), validada en español y que presenta una alta sensibilidad para la identificación de trastornos conductuales en niños de 4–16 años, correlacionándose con la presencia de un diagnóstico psiquiátrico en el 93,6% entre los que se incluye el TDAH [50]. Otra herramienta que podría ser útil a nivel clínico para la identificación de alteraciones en las funciones ejecutivas es la EpiTrack Junior [51] o la Behavior Rating Inventory of Executive Function (BRIEF) [52].

## 4. Tratamiento del TDAH en Niños con Epilepsia

### 4.1 Tratamiento No Farmacológico

Los principales objetivos del tratamiento del TDAH en el paciente con epilepsia es mejorar los síntomas nucleares que lo definen (inatención, hiperactividad, impulsividad), mejorar la calidad de vida del paciente y su familia, así como prevenir la aparición de complicaciones o comorbilidades a mediano y largo plazo.

En aquellos pacientes con epilepsia y TDAH comórbido el abordaje terapéutico debe ser similar al de aquellos pacientes sin epilepsia (tratamiento multimodal, que es aquel que incluye medidas de psicoeducación, abordaje psicológico-conductual, pedagógico y farmacológico) [35].

En el caso de población con epilepsia es fundamental el abordaje de trastornos comórbidos, cuyo tratamiento mejoraría la sintomatología de TDAH y la propia epilepsia, como son los trastornos de sueño y las comorbilidades psiquiátricas [53]. Igualmente hay que considerar la importancia de la elección del MAC; teniendo en cuenta tanto la optimización de una terapia anticrisis racional, al igual que evitar MAC que se relacionen con especial afectación cognitiva (como el topiramato, el ácido valproico o los GABAérgicos, como el fenobarbital y las benzodiacepinas) [54].

### 4.2 Tratamiento Farmacológico del TDAH

#### 4.2.1 Metilfenidato

El metilfenidato es un fármaco estimulante del sistema nervioso central. Su principal mecanismo de acción consiste en aumentar la transmisión dopaminérgica y noradrenérgica en el córtex prefrontal y el cuerpo estriado, mediante la inhibición de los transportadores presinápticos Los transportadores presinápticos de dopamina (DAT)1 y noradrenalina (NET), respectivamente. También aumenta la actividad del transportador vesicular de monoaminas (VMAT), que transporta las aminas (por ejemplo, dopamina, norepinefrina, serotonina, histamina) al interior de las vesículas localizadas en las neuronas presinápticas, en preparación para su liberación posterior. Todas estas acciones del metilfenidato dan lugar a una mayor disponibilidad de dopamina y noradrenalina en el espacio extracelular. Por otra parte, el metilfenidato es un agonista de los receptores de serotonina receptor de serotonina tipo 1A (5HT1A) y de los receptores adrenérgicos α2 [55,56]. En modelos animales, el metilfenidato ha demostrado aumentar los niveles de histamina en la corteza prefrontal, un mecanismo que podría contribuir a la regulación de la atención y del control de impulsos. Sin embargo, la relevancia clínica de este hallazgo en humanos no ha sido completamente establecida y la mayor parte de la evidencia disponible procede de estudios preclínicos [57].

El metilfenidato está comercializado en España en diferentes presentaciones: forma de liberación inmediata, en cápsulas de liberación modificada pellets 50:50 (50% liberación inmediata, 50% liberación prolongada) y 30:70, y en la forma de liberación prolongada por sistema osmótico metilfenidato-OROS® 22:78. La dosis eficaz en niños suele estar entre 0,5–1,5 mg/kg/día, pero debe ajustarse individualmente, subiendo de forma gradual (Tabla 3).

**Tabla 3.** **Principales fármacos utilizados para el tratamiento del TDAH en España, incluyendo tanto estimulantes como no estimulantes (actualizado en marzo de 2026)**.

Tipo de fármaco	Nombre genérico	Nombre comercial	Presentaciones	Dosificación
				(Dosis máxima)
Estimulantes	Metilfenidato (Liberación Inmediata)	Rubifen®	Comprimidos de 5, 10 y 20 mg	Iniciar con 5 mg 1–2 veces al día. Incrementos semanales de 5–10 mg.
		Medicebran®		
				(60 mg)
	Metilfenidato (Liberación Modificada 50:50)	Medikinet®	Cápsulas de 5, 10, 20, 30, 40 y 50 mg	Iniciar con 5 mg de MF LI 1–2 veces al día o 10 mg de Medikinet 1 vez al día. Incrementos semanales de 5–10 mg. Adultos: Iniciar con 10 mg, incrementos semanales de 10 mg.
				(Niños: 60 mg; Adultos: 80 mg)
		Rubifen Prolong®	Cápsulas de 10, 20, 30, 40 y 60 mg	Iniciar con 20 mg. Incrementos semanales de 10–20 mg.
				(Niños: 60 mg; Adultos: 80 mg)
	Metilfenidato (Liberación Modificada 30:70)	Equasym®	Cápsulas de 10, 20, 30, 40 y 50 mg	Iniciar con 5 mg de MF LI 1–2 veces al día o 10 mg de Equasym 1 vez al día. Incrementos semanales de 5–10 mg.
				(Niños: 60 mg; Adultos: 80 mg)
	Metilfenidato (Liberación Prolongada OROS® 22:78)	Concerta®	Cápsulas de 18, 27, 36 y 54 mg	Iniciar con 18 mg. Incrementos semanales de 18 mg.
		Rubicrono®		
		Atenza®		(Niños: 54 mg; Adolescentes y adultos: 72 mg)
	Lisdesanfetamina (Liberación Prolongada)	Elvanse®	Cápsulas de 30, 50, y 70 mg	Iniciar con 30 mg. Incrementos de 20 mg.
				(70 mg)
No Estimulantes	Atomoxetina	Atamax®	Cápsulas de 10, 18, 25, 40, 60 y 80 mg	Niños: Iniciar con dosis 0,5 mg/kg. Incrementos quincenales hasta 1,2 mg/kg/día.
		Atomoxetina Cinfa®		
		Strattera®	4 mg/mL solución oral	
		Atomoxetina TecniGen®		
	Guanfacina	Intuniv®	Comprimidos de 1, 2, 3 y 4 mg	Iniciar con 1 mg/día. Incrementos semanales de 1 mg.
				(7 mg)
	Clonidina	Catapresan®	Comprimidos 0,15 mg	En ≤45 kg: iniciar con 0,05 mg/día antes de acostarse. Incrementos cada 3–7 días de dividiendo la dosis hasta cada 6 horas.
				(0,3 mg/día).
				En >45 kg: iniciar con 0,1 mg/día antes de acostarse. Incrementos cada 3–7 días dividiendo la dosis hasta cada 6 horas.
				(0,4 mg/día).

Las formulaciones disponibles, las dosis máximas autorizadas y la situación regulatoria pueden variar entre países. Antes de prescribir cualquiera de estos medicamentos, se recomienda consultar la ficha técnica vigente y las autorizaciones regulatorias nacionales correspondientes. Nota: Las dosis mostradas corresponden a las recomendaciones habitualmente utilizadas en España y deben individualizarse según la edad, el peso, la respuesta clínica y la tolerabilidad del paciente. Las indicaciones autorizadas, formulaciones disponibles y dosis máximas pueden variar entre países.

#### 4.2.2 Eficacia del Metilfenidato en Pacientes con TDAH y Epilepsia

Tanto las guías actuales como los recientes metaanálisis en red basados en 190 ensayos clínicos aleatorizados apoyan firmemente el uso de psicoestimulantes como el tratamiento más eficaz para tratar el TDAH, teniendo en cuenta tanto las opciones farmacológicas como las no farmacológicas [35].

##### 4.2.2.1 Evidencia en Población Pediátrica

Los primeros estudios de eficacia del metilfenidato en niños con TDAH y epilepsia se centraron en demostrar la mejoría en diferentes medidas de disfunción ejecutiva, que suele ser una característica propia de niños y adultos con TDAH, en especial la planificación, la flexibilidad cognitiva y el control inhibitorio [58,59]. En este sentido, a finales del siglo pasado se publicaron varias series de 10–13 niños con TDAH y epilepsia en las que se observaba cómo el metilfenidato mejoraba varias funciones ejecutivas medidas mediante diferentes test neuropsicológicos [60,61,62].

La mejoría de los síntomas de TDAH se ha demostrado posteriormente mediante el empleo de escalas, en diferentes estudios de muestras pequeñas como el estudio retrospectivo de Socanski et al. [63] sobre 14 niños con TDAH y epilepsia, en el que se objetivó una mejoría del 85% en la sintomatología nuclear del TDAH, tras administrar metilfenidato, o el ensayo fase 1 de Gonzalez-Heydrich et al. [64], en el que reportaron mejoría de los síntomas nucleares del TDAH en el grupo tratado con metilfenidato-OROS® respecto al grupo placebo.

La eficacia del metilfenidato en el tratamiento del TDAH en niños con epilepsia se ha demostrado fundamentalmente en dos estudios abiertos con tamaños muestrales considerables. El primero de Gucuyener et al. [65] en el que evaluaron a 57 niños con epilepsia y 62 con anomalías electroencefalográficas, encontrando que el metilfenidato reducía significativamente los síntomas del TDAH tanto en la escala de Conners (*p* < 0,05) como en la ADHD Rating Scale (*p* < 0,001). Asimismo, en un estudio observacional multicéntrico realizado en Francia por Rheims et al. [66], que incluía a 167 niños con epilepsia de los cuales el 46% tenía epilepsia activa y el 26% estaba en tratamiento con más de dos medicamentos antiepilépticos, los resultados mostraron que aquellos que recibieron metilfenidato experimentaron una mejora significativa en los síntomas del TDAH según la ADHD Rating Scale, en comparación con los que no recibieron psicoestimulantes (75% frente a 42%; *p* < 0,001).

##### 4.2.2.2 Evidencia en Población Adulta

Aunque hay menos evidencia en adultos, destaca el estudio de Adams et al. [67]en el que realizan un ensayo doble ciego con grupo placebo en 28 adultos con TDAH y epilepsia, con una media de edad de 36,4 años y una media de 2,8 crisis al mes, en el que demostraron una mejora en el Test de ejecución continua de Conners, que evalúa la atención sostenida en el grupo tratado (*p* < 0,002).

En resumen, aunque se desconoce si la eficacia del metilfenidato varía según las etiologías y los diferentes síndromes epilépticos, se considera que la eficacia del metilfenidato en la población pediátrica con epilepsia es del 65%–85% [35], similar a la de población que no padece epilepsia. No obstante, estos resultados proceden de poblaciones heterogéneas y la evidencia disponible en pacientes con epilepsia farmacorresistente y encefalopatías epilépticas y del desarrollo continúa siendo limitada. La evidencia en adultos es más limitada, aunque los estudios disponibles apuntan en la misma dirección [66,67]. De hecho, en una reciente revisión de la Cochrane se concluyó que existía suficiente evidencia de que el metilfenidato era eficaz en población con TDAH y epilepsia [54]. Asimismo, en las últimas recomendaciones del Grupo de Comorbilidades de la ILAE se reportó un grado de recomendación de nivel B para el uso de metilfenidato en niños con epilepsia y TDAH, mientras que la atomoxetina y las anfetaminas fueron clasificadas como nivel U [41].

#### 4.2.3 Eficacia del Metilfenidato la Calidad de Vida y las Alteraciones Cognitivas 

Los pacientes con epilepsia presentan con frecuencia diferentes trastornos cognitivos, que van más allá de las funciones ejecutivas y los síntomas de inatención clásicamente descriptos en esta población. Estas alteraciones pueden mejorar tanto optimizando el control de las crisis mediante politerapia racional, como controlando los efectos secundarios de los MAC, y mediante estimulación o rehabilitación cognitiva [68].

Por su mecanismo de acción múltiple, el metilfenidato podría teóricamente contribuir a la mejora cognitiva y de memoria en los pacientes con epilepsia. Aparte de la mejora mediada por sus efectos noradrenérgicos y dopaminérgicos que regulan la actividad y la comunicación entre la corteza prefrontal ventrolateral y la formación del hipocampo, el metilfenidato también aumentaría los niveles extracelulares de histamina, la cual juega un papel fundamental en el estado de alerta, la memoria y el aprendizaje [57].

##### 4.2.3.1 Población Pediátrica

El metilfenidato se emplea para mejorar de forma global la disfunción cognitiva presente en determinadas patologías como la depresión mayor y diferentes enfermedades neurodegenerativas [69]. Sin embargo, una revisión narrativa concluyó que, aunque mejorara ciertos dominios cognitivos en personas con TDAH, podría no mejorar otros aspectos, como la distraibilidad y la planificación [70].

No obstante, un metaanálisis que examinó los efectos cognitivos del metilfenidato en niños y adolescentes con TDAH en 60 estudios, encontró efectos positivos estadísticamente significativos y consistentes no sólo en la memoria de trabajo sino en otros aspectos como el tiempo de reacción e inhibición de la respuesta [71].

##### 4.2.3.2 Población Adulta

Igualmente, diferentes estudios tanto en niños como en población adulta, han mostrado efectos positivos en los procesos de memoria especialmente en la memoria declarativa y viso-espacial [72].

La calidad de vida en estos pacientes se correlaciona de forma positiva con la puntuación de los síntomas de TDAH. Se sabe que los pacientes con epilepsia y TDAH presentan peores puntuaciones en comparación con los pacientes con epilepsia sin TDAH tanto en las áreas de bienestar físico, autoestima, relaciones familiares y sociales, como en el funcionamiento escolar. En esta misma línea, los resultados de una encuesta realizada a 1361 adultos con epilepsia activa pusieron en evidencia una peor calidad de vida cuando asociaban síntomas de TDAH [73]. En este contexto, el tratamiento con metilfenidato ha demostrado mejorar la calidad de vida en niños y adultos con TDAH y diferentes tipos de epilepsia [35,67,74].

#### 4.2.4 Seguridad de Metilfenidato en Pacientes Con Epilepsia

Aunque inicialmente los psicoestimulantes, en concreto los derivados anfetamínicos, fueron considerados potencialmente como MACs por la teoría de que la señalización del receptor DA pudiera suprimir la actividad convulsiva, las propiedades del metilfenidato como estimulante del SNC causaron preocupación entre los clínicos, ante la posibilidad de que pudiera aumentar el riesgo de presentar crisis al disminuir el umbral convulsivo [60]. De hecho, la información de diferentes fichas técnicas advierte que el metilfenidato “debe utilizarse con precaución en pacientes con epilepsia” y las agencias reguladoras norteamericanas y europeas advierten que “los estimulantes pueden reducir el umbral epileptógeno y deben suspenderse si aumenta la frecuencia de las convulsiones”.

##### 4.2.4.1 Seguridad en Población Pediátrica

Esta alarma se incrementó tras la publicación de casos aislados de niños sin epilepsia, en los cuales el inicio del metilfenidato, en muchos de ellos en combinación con sertralina, se relacionó con la aparición de crisis [75]. Hemmer et al. [76] alertaron de este posible riesgo de aumento de la actividad epileptiforme en un estudio en el que se realizaron EEGs seriados en un grupo de 234 niños sin epilepsia, y en el que concluyeron que el uso de metilfenidato podría provocar crisis en niños sin epilepsia (incidencia 0,6%); especialmente entre aquellos casos que presentaban actividad epiléptica intercrítica previa (incidencia 10%). Posteriormente este riesgo no se ha confirmado en dos grandes estudios observacionales. Tanto en un estudio realizado en Hong Kong en el que se siguieron a 29.604 niños y adultos jóvenes con diagnóstico de TDAH que tomaban metilfenidato, como en el realizado en Estados Unidos en donde la población seguida ascendió a más de 800.000 personas [77], no objetivaron que el tratamiento con metilfenidato produjera un aumento del riesgo de crisis [4]. Esta alarma fue mayor en aquellos pacientes con epilepsia.

Un único estudio prospectivo de una pequeña muestra de 33 niños con TDAH y epilepsia en el que se comparaba la frecuencia de crisis entre aquellos que iniciaron tratamiento con metilfenidato-OROS *versus* placebo, reportaron un aumento del riesgo de crisis, el cual era directamente proporcional a la dosis de metilfenidato empleada: 0,53 crisis/100 días en el grupo placebo, 1,12 crisis/100 días en aquellos que tomaron 36 mg y 2,30 crisis/100 días en el grupo de 54 mg (*p* < 0,001). Sin embargo, los autores indicaron que habría que comprobar estos resultados en nuevos estudios debido a las importantes limitaciones de su estudio: muestra pequeña y heterogénea, así como baja frecuencia de crisis basal (el criterio de inclusión fue haber tenido una crisis en los 5 años previos) [64].

Las limitaciones principales de todos estos estudios es que las muestras eran pequeñas, no se analizaron otros factores de riesgo convulsivo y la mayoría de los autores concluyeron que serían necesarios más estudios que confirmaran esta relación de metilfenidato como causante de crisis, ya que sus estudios no aportaban suficiente evidencia de causalidad [60,64,74].

Posteriormente se han publicado hasta 9 estudios clase II y 10 estudios clase III, que incluyen entre 11 y 167 pacientes, en los cuales no se encontró que el metilfenidato pudiera causar un aumento en la frecuencia o en la intensidad de las crisis ni en niños [64,65,66,74].

##### 4.2.4.2 Seguridad en Población Adulta

Park et al. [78] publicaron un estudio retrospectivo de 105 adolescentes y adultos jóvenes con epilepsia, en el que observaron que el uso de metilfenidato produjo un aumento de las crisis en un 20%. Estos datos se comprobaron en diferentes estudios posteriores en población adulta [67,74,79].

##### 4.2.4.3 Seguridad en Epilepsia Fármaco-Resistente

En el caso de la epilepsia refractaria, la mayoría de los estudios demostraron que el tratamiento con metilfenidato no aumentaba el riesgo de convulsiones en este tipo de pacientes. Sin embargo, hay dos estudios con una muestra pequeña que sugieren una cierta precaución. En el primero de ellos, Koneski et al. [80] observaron un aumento de la frecuencia de crisis a los 6 meses, en 2 de 24 pacientes, pero con una reducción de la gravedad de las crisis en el conjunto de la muestra. Posteriormente, en un estudio similar, Santos et al. [81] reportaron que en 4 de 22 niños con epilepsia refractaria presentaron un aumento de la frecuencia de crisis a los tres meses de iniciado el tratamiento con metilfenidato.

##### 4.2.4.4 Conclusiones de Seguridad

En los últimos años han aparecido varios estudios poblacionales que han aportado información adicional relevante sobre la seguridad del metilfenidato en población con epilepsia. En el Registro Sueco de Medicamentos Prescritos entre 2006–2013, 995 pacientes pediátricos con epilepsia redujeron la frecuencia de crisis en las primeras 24 semanas tras el inicio de tratamiento farmacológico del TDAH (HR 0,73; IC 95%: 0,57–0,94) [3]. Igualmente, un estudio retrospectivo en USA (entre 1999 y 2010), en el que se compararon un grupo de 18.166 niños con epilepsia y TDAH que habían iniciado tratamiento con estimulantes versus un grupo de 54.197 niños con TDAH sin tratamiento con psicoestimulantes; no encontraron una mayor tasa de hospitalización relacionada con crisis epilépticas (HR 0,95; IC 95%: 0,83–1,09 y HR 0,99; IC 95%:0,85–1,15) [1]. Asimismo, en ninguno de los estudios se identificó que la dosis de metilfenidato o la etiología, se asociara a un mayor riesgo de crisis epilépticas. Por todo ello, tanto las últimas revisiones de la Cochrane como las recomendaciones del Grupo de Comorbilidades de la ILAE, consideran al metilfenidato como seguro, con un nivel de recomendación B [41,54].

La evidencia actualmente disponible no permite establecer conclusiones sólidas sobre posibles diferencias en el riesgo según el síndrome epiléptico, la etiología o el grado de control de las crisis. Aunque el metilfenidato parece ser seguro en la mayoría de los pacientes con epilepsia, se recomienda una monitorización más estrecha e individualizada en aquellos con epilepsia farmacorresistente, empeoramiento reciente de las crisis o encefalopatías epilépticas y del desarrollo. En estos pacientes, la indicación de metilfenidato debe individualizarse tras valorar cuidadosamente el balance beneficio-riesgo y realizar un seguimiento clínico estrecho.

Dado que la mayoría de los pacientes con epilepsia presentan fluctuaciones espontáneas en la frecuencia de crisis, la aparición de un empeoramiento tras iniciar metilfenidato no implica necesariamente una relación causal. Antes de atribuir dicho empeoramiento al fármaco, deben descartarse otros factores potencialmente contribuyentes. Los autores proponen un algoritmo de manejo en caso de sospecha de empeoramiento debido a metilfenidato (Fig. 2).

**Fig. 2.** **Recomendaciones ante la sospecha de empeoramiento de epilepsia por metilfenidato**.

La evaluación de la seguridad del metilfenidato en pacientes con epilepsia debe considerar otros efectos adversos potencialmente relevantes. Entre ellos destacan la disminución del apetito, la pérdida de peso, el insomnio y otros trastornos del sueño. Este último aspecto resulta especialmente importante en pacientes con epilepsia, dado que la privación de sueño puede favorecer la aparición de crisis. Por otra parte, el metilfenidato presenta un potencial relativamente bajo de interacciones farmacocinéticas clínicamente relevantes con la mayoría de los medicamentos anticrisis. No obstante, debido a la frecuente politerapia en pacientes con epilepsia, se recomienda una monitorización individualizada tras el inicio o ajuste del tratamiento.

## 5. Limitaciones

Este documento presenta varias limitaciones que deben ser consideradas al interpretar sus recomendaciones. En primer lugar, se trata de una revisión narrativa de la literatura y no de una revisión sistemática, por lo que, aunque se realizó una búsqueda amplia de la evidencia científica disponible, no se siguió una metodología formal de selección y evaluación de estudios basada en criterios *Preferred Reporting Items for Systematic Reviews and Meta-Analyses* (PRISMA). Por ello, las recomendaciones propuestas deben interpretarse como una guía práctica basada en la mejor evidencia científica disponible y en el consenso de expertos, más que como recomendaciones derivadas de una revisión sistemática con evaluación formal de la calidad de la evidencia.

En segundo lugar, las recomendaciones propuestas se fundamentan tanto en la evidencia publicada como en el consenso de expertos. Aunque el panel estuvo integrado por neuropediatras de cinco hospitales españoles con amplia experiencia en epilepsia y trastornos del neurodesarrollo, no se empleó una metodología Delphi formal ni un sistema estructurado de votación para cuantificar el grado de acuerdo alcanzado.

Asimismo, la calidad de la evidencia disponible continúa siendo limitada. La mayoría de los estudios publicados sobre el uso de metilfenidato en pacientes con epilepsia y TDAH son observacionales, retrospectivos o cuentan con tamaños muestrales reducidos. Existen pocos ensayos clínicos controlados y aleatorizados específicamente diseñados para evaluar la eficacia y seguridad de este tratamiento en esta población. Además, la evidencia disponible no permite establecer conclusiones sólidas sobre posibles diferencias en la seguridad según el síndrome epiléptico, la etiología o el grado de control de las crisis.

Otra limitación relevante es la heterogeneidad de los pacientes incluidos en los estudios disponibles, que abarcan diferentes edades, tipos de epilepsia, etiologías, grados de control de crisis y tratamientos anticrisis concomitantes, dificultando la extrapolación de los resultados a subgrupos específicos de pacientes.

Por último, aunque se han incluido datos procedentes tanto de población pediátrica como adulta para proporcionar una visión más completa de la evidencia disponible, la mayor parte de los estudios se han realizado en niños y adolescentes. En consecuencia, algunas recomendaciones aplicables a la población adulta se sustentan en un menor volumen de evidencia y deben interpretarse con cautela hasta que se disponga de estudios específicos de mayor calidad metodológica.

## 6. Recomendaciones del Grupo de Expertos y Conclusión

A pesar de la preocupación que existe desde hace tiempo sobre la seguridad del uso de medicamentos estimulantes en pacientes con epilepsia, las pruebas de un riesgo aumentado de convulsiones asociadas al uso de fármacos para el TDAH son limitadas y basadas principalmente en reportes de series de casos, mientras que las grandes series y registros de pacientes poblacionales, sugieren que el metilfenidato no sólo no empeora la incidencia de las crisis sino que incluso puede disminuir el riesgo de que estas se produzcan [41,54].

Debido a que el TDAH produce un impacto adverso significativo en el funcionamiento académico, social y cognitivo del paciente con epilepsia y sus familiares, su tratamiento resulta de crucial importancia.

Los autores de esta revisión son conscientes de que son necesarios estudios aleatorizados frente a placebo, que permitan aumentar la evidencia disponible acerca de la eficacia y especialmente la seguridad del metilfenidato en población con epilepsia y TDAH [53]. La evidencia disponible es especialmente sólida en población pediátrica, mientras que los datos en adultos son más limitados pero globalmente concordantes respecto a la eficacia y seguridad del metilfenidato.

A pesar de la necesidad de mejorar la evidencia disponible acerca del tratamiento farmacológico del TDAH en niños y adultos con epilepsia, el tratamiento integral del paciente con epilepsia ha de incluir el tratamiento de sus comorbilidades, incluyendo el TDAH. En este sentido, mientras que el metilfenidato es un fármaco que ha demostrado ser eficaz para tratar la sintomatología TDAH en niños y adultos con epilepsia (similar al de la población sin epilepsia), evidencia disponible no sugiere un aumento clínicamente relevante de la frecuencia de crisis epilépticas en la mayoría de los pacientes con epilepsia tratados con metilfenidato, aunque no puede excluirse por completo un incremento del riesgo en determinadas poblaciones para las que la evidencia disponible sigue siendo limitada.

Por todo ello, este grupo de expertos considera que el metilfenidato puede ser una opción terapéutica en niños y adultos con epilepsia y TDAH cuando exista una indicación clínica, tras una adecuada valoración diagnóstica y la optimización del tratamiento de la epilepsia, sus comorbilidades y las intervenciones no farmacológicas. La decisión de iniciar tratamiento debe individualizarse, especialmente en pacientes con epilepsia activa, epilepsia farmacorresistente, empeoramiento reciente de las crisis o encefalopatías epilépticas y del desarrollo (Fig. 1).

## Contribuciones de los Autores

CF y VSI: Conceptualización, metodología, investigación, redacción-borrador original, y redacción-revisión y edición. MER, EGA, JLP, SAA, IMD: Conceptualización, metodología, y redacción-revisión y edición. Todos los autores han leído y aprobado la versión final del manuscrito. Asimismo, todos han participado de manera suficientemente significativa en el trabajo y han aceptado asumir la responsabilidad de todos los aspectos del mismo.

## Aprobación Ética y Consentimiento Informado

No aplicable.

## Financiación

Esta investigación no recibió financiación externa.

## References

[1] Liu X, Carney PR, Bussing R, Segal R, Cottler LB, Winterstein AG (2018). Stimulants Do Not Increase the Risk of Seizure-Related Hospitalizations in Children with Epilepsy. *Journal of Child and Adolescent Psychopharmacology*.

[2] McAfee AT, Landon J, Jones M, Bangs ME, Acharya N, Hornbuckle K (2013). A cohort study of the risk of seizures in a pediatric population treated with atomoxetine or stimulant medications. *Pharmacoepidemiology and Drug Safety*.

[3] Brikell I, Chen Q, Kuja-Halkola R, D’Onofrio BM, Wiggs KK, Lichtenstein P (2019). Medication treatment for attention-deficit/hyperactivity disorder and the risk of acute seizures in individuals with epilepsy. *Epilepsia*.

[4] Wiggs KK, Chang Z, Quinn PD, Hur K, Gibbons R, Dunn D (2018). Attention-deficit/hyperactivity disorder medication and seizures. *Neurology*.

[5] WHO (2005). The use of the WHO-UMC system for standardised case causality assessment. https://www.who.int/docs/default-source/medicines/pharmacovigilance/whocausality-assessment.pdf.

[6] Polanczyk G, de Lima MS, Horta BL, Biederman J, Rohde LA (2007). The worldwide prevalence of ADHD: a systematic review and metaregression analysis. *The American Journal of Psychiatry*.

[7] Thomas R, Sanders S, Doust J, Beller E, Glasziou P (2015). Prevalence of attention-deficit/hyperactivity disorder: a systematic review and meta-analysis. *Pediatrics*.

[8] Catalá-López F, Peiró S, Ridao M, Sanfélix-Gimeno G, Gènova-Maleras R, Catalá MA (2012). Prevalence of attention deficit hyperactivity disorder among children and adolescents in Spain: a systematic review and meta-analysis of epidemiological studies. *BMC Psychiatry*.

[9] Fisher RS, Acevedo C, Arzimanoglou A, Bogacz A, Cross JH, Elger CE (2014). ILAE official report: a practical clinical definition of epilepsy. *Epilepsia*.

[10] Sadek J (2019). Clinician’s Guide to ADHD Comorbidities in Children and Adolescents. *Case Studios*.

[11] Wang S, Yao B, Zhang H, Xia L, Yu S, Peng X (2023). Comorbidity of epilepsy and attention-deficit/hyperactivity disorder: a systematic review and meta-analysis. *Journal of Neurology*.

[12] Kaufmann R, Goldberg-Stern H, Shuper A (2009). Attention-deficit disorders and epilepsy in childhood: incidence, causative relations and treatment possibilities. *Journal of Child Neurology*.

[13] Dunn DW, Austin JK, Harezlak J, Ambrosius WT (2003). ADHD and epilepsy in childhood. *Developmental Medicine and Child Neurology*.

[14] Wolraich ML, Hagan JF, Allan C, Chan E, Davison D, Earls M (2019). Clinical Practice Guideline for the Diagnosis, Evaluation, and Treatment of Attention-Deficit/Hyperactivity Disorder in Children and Adolescents. *Pediatrics*.

[15] Kooij JJS, Buitelaar JK, van den Oord EJ, Furer JW, Rijnders CAT, Hodiamont PPG (2005). Internal and external validity of attention-deficit hyperactivity disorder in a population-based sample of adults. *Psychological Medicine*.

[16] Aaberg KM, Bakken IJ, Lossius MI, Lund Søraas C, Håberg SE, Stoltenberg C (2016). Comorbidity and Childhood Epilepsy: A Nationwide Registry Study. *Pediatrics*.

[17] Bertelsen EN, Larsen JT, Petersen L, Christensen J, Dalsgaard S (2016). Childhood Epilepsy, Febrile Seizures, and Subsequent Risk of ADHD. *Pediatrics*.

[18] Brikell I, Ghirardi L, D’Onofrio BM, Dunn DW, Almqvist C, Dalsgaard S (2018). Familial Liability to Epilepsy and Attention-Deficit/Hyperactivity Disorder: A Nationwide Cohort Study. *Biological Psychiatry*.

[19] Rheims S, Auvin S (2021). Attention deficit/hyperactivity disorder and epilepsy. *Current Opinion in Neurology*.

[20] Austin JK, Harezlak J, Dunn DW, Huster GA, Rose DF, Ambrosius WT (2001). Behavior problems in children before first recognized seizures. *Pediatrics*.

[21] Dunn DW, Austin JK, Perkins SM (2009). Prevalence of psychopathology in childhood epilepsy: categorical and dimensional measures. *Developmental Medicine and Child Neurology*.

[22] Williams AE, Giust JM, Kronenberger WG, Dunn DW (2016). Epilepsy and attention-deficit hyperactivity disorder: links, risks, and challenges. *Neuropsychiatric Disease and Treatment*.

[23] Bechtel N, Kobel M, Penner IK, Specht K, Klarhöfer M, Scheffler K (2012). Attention-deficit/hyperactivity disorder in childhood epilepsy: a neuropsychological and functional imaging study. *Epilepsia*.

[24] Pascual-Morena C, Martínez-García I, Lucerón-Lucas-Torres M, Rodríguez-Gutiérrez E, Reynolds-Cortez V, Moreno-Charco E (2025). Prevalence of neurodevelopmental and psychiatric disorders in Noonan syndrome: a systematic review and meta-analysis. *European Journal of Pediatrics*.

[25] Ekinci O, Okuyaz Ç, Erdoğan S, Gunes S, Ekinci N, Kalınlı M (2017). Attention-Deficit Hyperactivity Disorder (ADHD) in Epilepsy and Primary ADHD: Differences in Symptom Dimensions and Quality of Life. *Journal of Child Neurology*.

[26] Davis SM, Katusic SK, Barbaresi WJ, Killian J, Weaver AL, Ottman R (2010). Epilepsy in children with attention-deficit/hyperactivity disorder. *Pediatric Neurology*.

[27] He Z, Yang X, Li Y, Zhao X, Li J, Li B (2024). Attention-deficit/hyperactivity disorder in children with epilepsy: A systematic review and meta-analysis of prevalence and risk factors. *Epilepsia Open*.

[28] Hermann B, Jones J, Dabbs K, Allen CA, Sheth R, Fine J (2007). The frequency, complications and aetiology of ADHD in new onset paediatric epilepsy. *Brain: a Journal of Neurology*.

[29] Hesdorffer DC, Ludvigsson P, Olafsson E, Gudmundsson G, Kjartansson O, Hauser WA (2004). ADHD as a risk factor for incident unprovoked seizures and epilepsy in children. *Archives of General Psychiatry*.

[30] Sherman EMS, Slick DJ, Connolly MB, Eyrl KL (2007). ADHD, neurological correlates and health-related quality of life in severe pediatric epilepsy. *Epilepsia*.

[31] Schatz DB, Rostain AL (2006). ADHD with comorbid anxiety: a review of the current literature. *Journal of Attention Disorders*.

[32] Kwong KL, Lam D, Tsui S, Ngan M, Tsang B, Lai TS (2016). Self-esteem in adolescents with epilepsy: Psychosocial and seizure-related correlates. *Epilepsy & Behavior: E&B*.

[33] Chen Q, Sjölander A, Runeson B, D’Onofrio BM, Lichtenstein P, Larsson H (2014). Drug treatment for attention-deficit/hyperactivity disorder and suicidal behaviour: register based study. *BMJ (Clinical Research Ed.)*.

[34] Kanner AM (2016). Management of psychiatric and neurological comorbidities in epilepsy. *Nature Reviews. Neurology*.

[35] National Institute for Health and Clinical Excellence (2018). Attention deficit hyperactivity disorder: diagnosis and management. https://www.nice.org.uk/guidance/ng87.

[36] Reilly C, Atkinson P, Das KB, Chin RFMC, Aylett SE, Burch V (2014). Neurobehavioral comorbidities in children with active epilepsy: a population-based study. *Pediatrics*.

[37] Gonzalez-Heydrich J, Hamoda HM, Luna L, Rao S, McClendon J, Rotella P (2012). Elevated rates of ADHD in mothers of children with comorbid ADHD and epilepsy. *Neuropsychiatry*.

[38] McCusker CG, Kennedy PJ, Anderson J, Hicks EM, Hanrahan D (2002). Adjustment in children with intractable epilepsy: importance of seizure duration and family factors. *Developmental Medicine and Child Neurology*.

[39] Aricò M, Arigliani E, Giannotti F, Romani M (2020). ADHD and ADHD-related neural networks in benign epilepsy with centrotemporal spikes: A systematic review. *Epilepsy & Behavior: E&B*.

[40] Eapen M, Iype M, Saradakutty G, Jayan BB, Sreedharan M, Ahamed S (2024). Childhood Absence Epilepsy- Electroclinical Profile and Prevalence of Attention-Deficit/Hyperactivity Disorder Among a Cohort of 47 Children. *Pediatric Neurology*.

[41] Auvin S, Wirrell E, Donald KA, Berl M, Hartmann H, Valente KD (2018). Systematic review of the screening, diagnosis, and management of ADHD in children with epilepsy. Consensus paper of the Task Force on Comorbidities of the ILAE Pediatric Commission. *Epilepsia*.

[42] Li J, Liu T, Liu C, Deng J, Wu S, Kuang S (2026). Treatment of pediatric epilepsy. *Pediatric Investigation*.

[43] Furones García M, García Peñas JJ, González Alguacil E, Moreno Cantero T, Ruiz Falcó ML, Cantarín Extremera V (2024). Sleep disorders in children with epilepsy. *Neurologia*.

[44] Kral MC, Lally MD, Boan AD (2016). Identification of ADHD in youth with epilepsy. *Journal of Pediatric Rehabilitation Medicine*.

[45] American Psychiatric Association (2013). Diagnostic and statistical manual of mental disorders: DSM-5™ (5th edn).

[46] Loutfi KS, Carvalho AM, Lamounier JA, Nascimento JDA (2011). ADHD and epilepsy: contributions from the use of behavioral rating scales to investigate psychiatric comorbidities. *Epilepsy & Behavior: E&B*.

[47] Scheffer IE, Berkovic S, Capovilla G, Connolly MB, French J, Guilhoto L (2017). ILAE classification of the epilepsies: Position paper of the ILAE Commission for Classification and Terminology. *Epilepsia*.

[48] Achenbach TM, Rescorla LA (2001). Manual for the ASEBA School-Age Forms & Profiles.

[49] Hesdorffer DC, Baldin E, Caplan R, Berg AT (2014). How do we measure psychiatric diagnoses? Implications of the choice of instruments in epilepsy. *Epilepsy & Behavior: E&B*.

[50] Alfstad KÅ, Torgersen H, Van Roy B, Hessen E, Hansen BH, Henning O (2016). Psychiatric comorbidity in children and youth with epilepsy: An association with executive dysfunction?. *Epilepsy & Behavior: E&B*.

[51] Helmstaedter C, Schoof K, Rossmann T, Reuner G, Karlmeier A, Kurlemann G (2010). Introduction and first validation of EpiTrack Junior, a screening tool for the assessment of cognitive side effects of antiepileptic medication on attention and executive functions in children and adolescents with epilepsy. *Epilepsy & Behavior: E&B*.

[52] McCandless S, O’ Laughlin L (2007). The Clinical Utility of the Behavior Rating Inventory of Executive Function (BRIEF) in the diagnosis of ADHD. *Journal of Attention Disorders*.

[53] Leeman-Markowski BA, Adams J, Martin SP, Devinsky O, Meador KJ (2021). Methylphenidate for attention problems in epilepsy patients: Safety and efficacy. *Epilepsy & Behavior: E&B*.

[54] Eaton C, Yong K, Walter V, Mbizvo GK, Rhodes S, Chin RF (2022). Stimulant and non-stimulant drug therapy for people with attention deficit hyperactivity disorder and epilepsy. *The Cochrane Database of Systematic Reviews*.

[55] Faraone SV (2018). The pharmacology of amphetamine and methylphenidate: Relevance to the neurobiology of attention-deficit/hyperactivity disorder and other psychiatric comorbidities. *Neuroscience and Biobehavioral Reviews*.

[56] Wolraich ML, Doffing MA (2004). Pharmacokinetic considerations in the treatment of attention-deficit hyperactivity disorder with methylphenidate. *CNS Drugs*.

[57] Horner WE, Johnson DE, Schmidt AW, Rollema H (2007). Methylphenidate and atomoxetine increase histamine release in rat prefrontal cortex. *European Journal of Pharmacology*.

[58] Pietrzak RH, Mollica CM, Maruff P, Snyder PJ (2006). Cognitive effects of immediate-release methylphenidate in children with attention-deficit/hyperactivity disorder. *Neuroscience and Biobehavioral Reviews*.

[59] Hervey AS, Epstein JN, Curry JF (2004). Neuropsychology of adults with attention-deficit/hyperactivity disorder: a meta-analytic review. *Neuropsychology*.

[60] Gross-Tsur V, Manor O, van der Meere J, Joseph A, Shalev RS (1997). Epilepsy and attention deficit hyperactivity disorder: is methylphenidate safe and effective?. *The Journal of Pediatrics*.

[61] Feldman H, Crumrine P, Handen BL, Alvin R, Teodori J (1989). Methylphenidate in children with seizures and attention-deficit disorder. *American Journal of Diseases of Children (1960)*.

[62] Semrud-Clikeman M, Wical B (1999). Components of attention in children with complex partial seizures with and without ADHD. *Epilepsia*.

[63] Socanski D, Aurlien D, Herigstad A, Thomsen PH, Larsen TK (2013). Epilepsy in a large cohort of children diagnosed with attention deficit/hyperactivity disorders (ADHD). *Seizure*.

[64] Gonzalez-Heydrich J, Whitney J, Waber D, Forbes P, Hsin O, Faraone SV (2010). Adaptive phase I study of OROS methylphenidate treatment of attention deficit hyperactivity disorder with epilepsy. *Epilepsy & Behavior: E&B*.

[65] Gucuyener K, Erdemoglu AK, Senol S, Serdaroglu A, Soysal S, Kockar AI (2003). Use of methylphenidate for attention-deficit hyperactivity disorder in patients with epilepsy or electroencephalographic abnormalities. *Journal of Child Neurology*.

[66] Rheims S, Herbillon V, Villeneuve N, Auvin S, Napuri S, Cances C (2016). ADHD in childhood epilepsy: Clinical determinants of severity and of the response to methylphenidate. *Epilepsia*.

[67] Adams J, Alipio-Jocson V, Inoyama K, Bartlett V, Sandhu S, Oso J (2017). Methylphenidate, cognition, and epilepsy: A 1-month open-label trial. *Epilepsia*.

[68] Witt JA, Helmstaedter C (2012). Should cognition be screened in new-onset epilepsies? A study in 247 untreated patients. *Journal of Neurology*.

[69] Prommer E (2012). Methylphenidate: established and expanding roles in symptom management. *The American Journal of Hospice & Palliative Care*.

[70] Advokat C (2010). What are the cognitive effects of stimulant medications? Emphasis on adults with attention-deficit/hyperactivity disorder (ADHD). *Neuroscience and Biobehavioral Reviews*.

[71] Coghill DR, Seth S, Pedroso S, Usala T, Currie J, Gagliano A (2014). Effects of methylphenidate on cognitive functions in children and adolescents with attention-deficit/hyperactivity disorder: evidence from a systematic review and a meta-analysis. *Biological Psychiatry*.

[72] Verster JC, Bekker EM, Kooij JJS, Buitelaar JK, Verbaten MN, Volkerts ER (2010). Methylphenidate significantly improves declarative memory functioning of adults with ADHD. *Psychopharmacology*.

[73] Ettinger AB, Ottman R, Lipton RB, Cramer JA, Fanning KM, Reed ML (2015). Attention-deficit/hyperactivity disorder symptoms in adults with self-reported epilepsy: Results from a national epidemiologic survey of epilepsy. *Epilepsia*.

[74] Moore JL, McAuley JW, Long L, Bornstein R (2002). An Evaluation of the Effects of Methylphenidate on Outcomes in Adult Epilepsy Patients. *Epilepsy & Behavior: E&B*.

[75] Schertz M, Steinberg T (2008). Seizures induced by the combination treatment of methylphenidate and sertraline. *Journal of Child and Adolescent Psychopharmacology*.

[76] Hemmer SA, Pasternak JF, Zecker SG, Trommer BL (2001). Stimulant therapy and seizure risk in children with ADHD. *Pediatric Neurology*.

[77] Man KKC, Lau WCY, Coghill D, Besag FMC, Cross JH, Ip P (2020). Association between methylphenidate treatment and risk of seizure: a population-based, self-controlled case-series study. *The Lancet. Child & Adolescent Health*.

[78] Park J, Choi HW, Yum MS, Ko TS, Shon SH, Kim HW (2018). Relationship Between Aggravation of Seizures and Methylphenidate Treatment in Subjects with Attention-Deficit/Hyperactivity Disorder and Epilepsy. *Journal of Child and Adolescent Psychopharmacology*.

[79] van der Feltz-Cornelis CM, Aldenkamp AP (2006). Effectiveness and safety of methylphenidate in adult attention deficit hyperactivity disorder in patients with epilepsy: an open treatment trial. *Epilepsy & Behavior: E&B*.

[80] Koneski JAS, Casella EB, Agertt F, Ferreira MG (2011). Efficacy and safety of methylphenidate in treating ADHD symptoms in children and adolescents with uncontrolled seizures: a Brazilian sample study and literature review. *Epilepsy & Behavior: E&B*.

[81] Santos K, Palmini A, Radziuk AL, Rotert R, Bastos F, Booij L (2013). The impact of methylphenidate on seizure frequency and severity in children with attention-deficit-hyperactivity disorder and difficult-to-treat epilepsies. *Developmental Medicine and Child Neurology*.
